# Diiron Aminocarbyne Complexes with NCE^−^ Ligands (E = O, S, Se)

**DOI:** 10.3390/molecules28073251

**Published:** 2023-04-05

**Authors:** Giulio Bresciani, Stefano Zacchini, Guido Pampaloni, Marco Bortoluzzi, Fabio Marchetti

**Affiliations:** 1 Department of Chemistry and Industrial Chemistry, University of Pisa, Via G. Moruzzi 13, I-56124 Pisa, Italy; 2 Interuniversity Consortium for Chemical Reactivity and Catalysis, CIRCC, Via Celso Ulpiani 27, I-70126 Bari, Italy; 3 Department of Industrial Chemistry “Toso Montanari”, University of Bologna, Viale Risorgimento 4, I-40136 Bologna, Italy; 4 Department of Molecular Science and Nanosystems, University of Venezia “Ca’ Foscari”, Via Torino 155, I-30170 Mestre, Italy

**Keywords:** organometallic chemistry, diiron complexes, aminocarbyne ligand, selenocyanate, substitution reactions

## Abstract

Diiron μ-aminocarbyne complexes [Fe_2_Cp_2_(NCMe)(CO)(μ-CO){μ-CN(Me)(R)}]CF_3_SO_3_ (R = Xyl, **[1a^NCMe^]CF_3_SO_3_**; R = Me, **[1b^NCMe^]CF_3_SO_3_**; R = Cy, **[1c^NCMe^]CF_3_SO_3_**; R = CH_2_Ph, **[1d^NCMe^]CF_3_SO_3_**), freshly prepared from tricarbonyl precursors **[1a–d]CF_3_SO_3_**, reacted with NaOCN (in acetone) and NBu_4_SCN (in dichloromethane) to give [Fe_2_Cp_2_(k*N*-NCO)(CO)(μ-CO){μ-CN(Me)(R)}] (R = Xyl, **2a**; Me, **2b**; Cy, **2c**) and [Fe_2_Cp_2_(k*N*-NCS)(CO)(μ-CO){μ-CN(Me)(CH_2_Ph)}], **3** in 67–81% yields via substitution of the acetonitrile ligand. The reaction of **[1a^NCMe^–1c^NCMe^]CF_3_SO_3_** with KSeCN in THF at reflux temperature led to the cyanide complexes [Fe_2_Cp_2_(CN)(CO)(μ-CO){μ-CNMe(R)}], **6a**–**c** (45–67%). When the reaction of **[1a^NCMe^]CF_3_SO_3_** with KSeCN was performed in acetone at room temperature, subsequent careful chromatography allowed the separation of moderate amounts of [Fe_2_Cp_2_(k*Se*-SeCN)(CO)(μ-CO){μ-CN(Me)(Xyl)}], **4a**, and [Fe_2_Cp_2_(k*N*-NCSe)(CO)(μ-CO){μ-CN(Me)(Xyl)}], **5a**. All products were fully characterized by elemental analysis, IR, and multinuclear NMR spectroscopy; moreover, the molecular structure of ***trans*-6b** was ascertained by single crystal X-ray diffraction. DFT calculations were carried out to shed light on the coordination mode and stability of the {NC*Se*-} fragment.

## 1. Introduction

Pseudohalides of the general formula {NCE}^−^ (E = O, S, Se) are ubiquitous and largely employed in coordination chemistry, and they may behave as ligands through either the nitrogen or chalcogen atom according to the nature of the metal center [1,2,3,4,5,6,7,8,9,10,11]. Metal binding of {NCO}^−^ through the nitrogen atom (isocyanate) is predominant over the *O*-coordination (cyanate), and activation of the isocyanate ligand by nucleophilic addition to the unsaturated carbon atom may be subsequently viable [12,13]. On the other hand, coordination linkage isomerism is often observed with complexes comprising an {NCE}^−^ ligand (E = S, Se) [14,15,16,17], and coordination switching from nitrogen (isothiocyanate) to sulfur (thiocyanate) may be achieved upon heating or UV irradiation [18].

A variety of selenocyanate/isoselenocyanate complexes have been synthesized [19,20,21,22], and thermogravimetric analyses on cobalt(II)-selenocyanate complexes evidenced stability up to 200 °C [23].

Notwithstanding, the metal coordination of the {NCSe}^−^ group might be unstable, resulting in decomposition into the cyanide ion and selenium atom; this reaction was previously reported with copper(II) acetate [24,25] and was exploited for catalytic purposes in cyanation reactions. Selenocyanate to cyanide degradation was also observed in human blood, and the ferric ion is typically involved in this process [26]. However, to the best of our knowledge, the clean isolation of a metal–cyanide derivative following activation of the coordinated {NCSe}^−^ moiety is a rare feature. Note that the reverse reaction, i.e., the cyanation of elemental selenium, is typically conducted to prepare selenocyanate compounds [27,28].

The {Fe_2_Cp_2_(CO)_x_} skeleton (x = 2–3) is a versatile scaffold for building organometallic architectures that take advantage of metal–metal cooperativity [29,30,31,32], and it can be used to explore novel reactivity patterns exploiting an earth-abundant metal element [33,34,35]. 

**Scheme 1:**
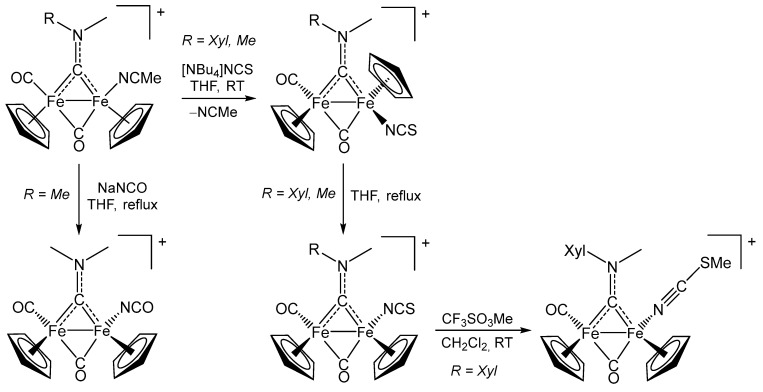
Synthesis of diiron μ-aminocarbyne complexes with isocyanate and isothiocyanate as the terminal ligands and *S*-methylation reaction. Xyl = 2,6-C_6_H_3_Me_2_.

In this framework, in the last 20 years, our research has focused on the chemistry of diiron complexes with a bridging aminocarbyne ligand [36,37,38], and we previously reported the synthesis of isocyanate and isothiocyanate derivatives from a labile acetonitrile precursor (Scheme 1) [39]. The reaction with tetrabutylammonium thiocyanate at room temperature selectively afforded the *N*-coordinated product with the Cp ligands mutually *trans* oriented with respect to the Fe–Fe axis, and then thermal conversion into the *cis* isomer was observed without affecting the isothiocyanate moiety. On the other hand, the installation of the isocyanate ligand proceeded at high temperature only. We also demonstrated in one case that methylation of the isothiocyanate ligand was straightforward, affording a thiomethyl-nitrile species. 

Here, we extended the chemistry of diiron aminocarbyne complexes with pseudohalide ligands, including reactions with a selenocyanate source. Structural and thermodynamic aspects were elucidated by means of DFT calculations.

## 2. Results and Discussion

**Scheme 2:**
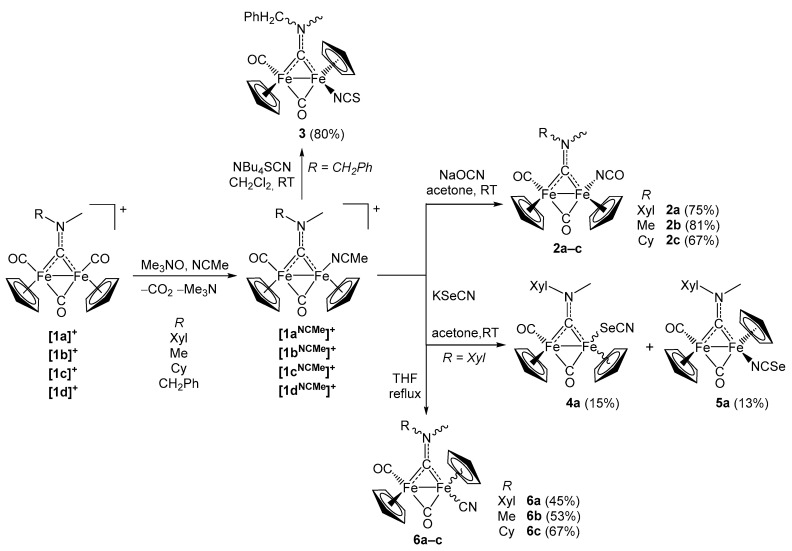
Installation of chalcogen cyanate ligands on diiron μ-aminocarbyne complexes and thermal selenocyanate to cyanide conversion (Xyl = 2,6–C_6_H_3_Me_2_; Cy = C_6_H_11_, cyclohexyl; Ph = C_6_H_5_). Yields in parentheses.

The diiron aminocarbyne complexes **[1a–d]CF_3_SO_3_** were prepared from commercially available [Fe_2_Cp_2_(CO)_4_] following the appropriate literature procedures (see Experimental). To evaluate the influence of the aminocarbyne ligand on the substitution reactions herein discussed, we considered a series of R substituents bearing different steric and electronic properties (Scheme 2). In fact, previous findings indicated that R may significantly affect the chemistry of this class of complexes [37]. Compounds **[1a–d]CF_3_SO_3_** were converted into the respective acetonitrile adducts [40] using the trimethylamine-*N*-oxide (TMNO) strategy, which is often reliable with cationic complexes based on the {M_2_Cp_2_(CO)_3_} core with M = Fe or Ru [41,42,43,44]. The resulting derivatives **[1a^NCMe^–1d^NCMe^]CF_3_SO_3_** were used in all cases as freshly prepared reactants. 

The reactions of **[1a^NCMe^–1c^NCMe^]CF_3_SO_3_** with sodium cyanate were performed in acetone at room temperature and afforded the neutral complexes **2a**–**c** in 67–81% yields directly as *cis* isomers, in alignment with the major stability of this configuration that is usually exhibited by complexes based on the [Fe_2_(CO)_2_Cp_2_] frame [36]. Thus, the IR and NMR spectra of the previously reported complex **2b** (see Scheme 1) matched the literature data [39]. The IR spectra of **2a** and **2c** (in CH_2_Cl_2_, 2300–1500 cm^−1^ spectral region) were similar to that of **2b**, consisting of three bands related to the coordinated cyanate ligand and the terminal and bridging carbonyl ligands (e.g., at 2242, 1986, and 1818 cm^−1^, respectively, in the case of **2a**). The major stability of *N*-coordination (compared to *O*-coordination) of the {NCO} moiety was as expected for a low-valent iron center [45,46,47]; otherwise, the preference for *O*-coordination of potential *N*- and *O*-donors is commonly observed with high-valent metal complexes [48,49,50]. Note that *O*-coordination towards the Fe^I^ center in related systems has been rarely observed and only as part of a coordination by means of multidentate hydrocarbyl ligands [51,52]. In **2c**, the band due to the μ-CN moiety fell at 1540 cm^−1^, in alignment with some double bond character (vide infra). In **2b**, the corresponding band was at 1578 cm^−1^.

The NMR spectra of **2b** displayed one set of resonances, while the NMR spectra of **2a** and **2c** contained two sets of resonances ascribable to the α and β isomers, differing in the orientation of the *N*-substituents with respect to the cyanate ligand. This kind of isomerism was previously reported for diiron and diruthenium complexes of the type [M_2_Cp_2_(L)(CO)(μ-CO){μ-CN(Me)(R)}]^0/+^ with R ≠ Me [36,37]. The α isomer (R pointing to NCO) was slightly prevalent with respect to the β isomer (R pointing to terminal CO) in the case of **2c** (α/β ratio = 1.3), whereas the bulkier xylyl group made the α form much more favorable in **2a** (α/β ratio = 4.5). Notably, that rotation of the amine group around the carbyne-nitrogen axis was inhibited due to the substantial C=N double bond character [36,37]. Ongoing from a to b, the *N*-Me resonance shifted to high fields by ca. 0.3 ppm in the ^1^H NMR spectra. The salient ^13^C NMR feature was provided by the carbyne carbon, resonating within the 329–338 ppm range [36,53].

Motivated by our interest in the chemistry of carbamato species [54,55,56] and based on the documented reactivity of isocyanates [12,57,58], we tested the reactivity of **2a,c** with a range of alcohols and amines, but no reaction occurred even under high-temperature conditions. The substantial inertness of the isocyanate ligand in **2a** was observed even towards strong electrophiles (i.e., methyl triflate and trimethylsilyl triflate).

The reaction of the benzyl-aminocarbyne complex **[1d^NCMe^]CF_3_SO_3_** with tetrabutylammonium thiocyanate was conducted in dichloromethane at room temperature and led to **3**, which was finally isolated in 80% yield. The IR spectrum of **3** (in CH_2_Cl_2_) displayed carbonyl absorptions at 1970 and 1810 cm^−1^, which were indicative of the *trans* configuration of the Cp ligands. Moreover, the {NCS} group was *N*-coordinated to the iron center, on account of an infrared absorption at 2114 cm^−1^. This value was very close to that previously detected in other {Fe-NCS} species [2,15,39]. The NMR spectra of **3** pointed out the occurrence of α/β isomerism, with the α isomer prevailing. The same behavior was observed when related methyl- and benzyl-aminocarbyne complexes were used (Scheme 1) [39].

The reactivity of the diiron aminocarbyne complexes with a selenium compound was investigated for the first time by allowing **[1a^NCMe^–1c^NCMe^]CF_3_SO_3_** to react with potassium selenocyanate in acetone. When these reactions were conducted at room temperature, complicated mixtures of products were afforded, including modest amounts of **6a**–**c**. Careful alumina chromatography on the mixture arising from **[1a^NCMe^]CF_3_SO_3_** allowed the separation of three components, which were spectroscopically analyzed and thus identified as complexes **4a** (15% yield), **5a** (13%), and **6a** (48%). Complexes **4a** and **5a** comprised *Se*- and *N*-coordinated selenocyanate ligands, respectively, while **6a** was a cyanide adduct. The infrared absorption for the pseudohalide ligand was detected at 2113 (**4a**) and 2109 cm^−1^ (**5a**); in general, the stretching vibration of a metal-coordinated {SeCN} group occurred at higher frequencies when it was *Se*-coordinated rather than *N*-coordinated [21,59,60,61,62]. The ^77^Se NMR spectra of **4a** and **5a** clearly pointed out the different coordinations of the {SeCN} moiety. Thus, two signals were recognized in the ^77^Se spectrum of **4a**, at −232.5 (major) and −246.9 ppm (minor) [63,64], while the ^77^Se spectrum of **5a** displayed a unique signal at −340.2 ppm. This picture was consistent with the literature data reported for other complexes and the general trend whereby ^77^Se NMR shielding increases from {M-SeCN} ongoing to {M-NCSe} [65]. The ^1^H and ^13^C NMR spectra of **4a** displayed two sets of resonances, attributed to *cis* and *trans* isomers, while the ^1^H and ^13^C NMR spectra of **5a** closely resembled those related to the homologous complex featuring a *cis* arrangement of the Cp rings and an *N*-coordinated NCS ligand [39]. In the ^13^C NMR spectrum of **4a**, the resonance for the selenocyanate ligand occurred above 128.7 ppm, whereas the iso-selenocyanate resonated at 108.4 ppm in the ^13^C NMR spectrum of **5a** [63,66,67]. The signal for the aminocarbyne carbon was upfield shifted in **5a** (340.4 ppm) compared to **4a** (345.4 ppm in the *trans* isomer), suggesting a different degree of back-donation from the diiron backbone to the carbyne in **4a** and **5a** [36]. Formation of *cis/trans* mixtures is believed to be consequent to rotation around the Fe–Fe bond (Adams–Cotton mechanism) [68,69], which is operative during the nitrile substitution process. In the majority of the cases, *trans* isomers based on the Fe_2_Cp_2_(CO)_x_ (x = 2, 3) scaffold are kinetic and less thermodynamically favored products, which might be observed due to a combination of electronic and steric effects [36,37,39,70]. In the case of **4a** and **5a**, stability studies revealed that the *trans* to *cis* route was not viable in boiling THF solution, whereas formation of cyanide complexes was observed (vide infra). Evidence for the formation of (iso)selenocyanate complexes (IR spectroscopy) was supplied by the room temperature reactions of [**1b,c^NCMe^]CF_3_SO_3_** with KSeCN, but attempts to isolate and characterize the products failed. 

**Figure 1:**
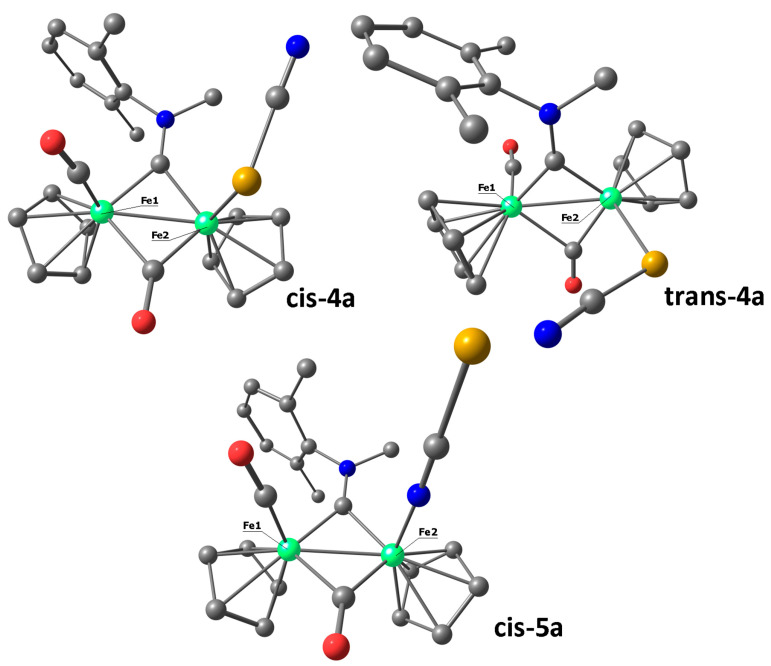
DFT-optimized structures of ***cis*-4a**, ***trans*-4a**, and ***cis*-5a** (C-PCM/PBEh-3c, acetone as continuous medium). Color map: Fe, green; Se, dark yellow; O, red; N, blue; C, grey; hydrogen atoms omitted for clarity. Selected computed bond lengths (Å): ***cis*-4a**, Fe1–C(CO) 1.766, Fe1–C(μ-CO) 1.994, Fe1–C(carbyne) 1.856, Fe1–C(Cp, average) 2.113, Fe2–Se 2.440, Fe2–C(μ-CO) 1.839, Fe2–C(carbyne) 1.855, Fe2–C(Cp, average) 2.117, Fe1–-Fe2 2.490, *Se*–C 1.813, C–N(SeCN) 1.159, C–N(carbyne) 1.298; ***trans*-4a**, Fe1–C(CO) 1.760, Fe1–C(μ-CO) 2.077, Fe1–C(carbyne) 1.842, Fe1–C(Cp, average) 2.121, Fe2–Se 2.449, Fe2–C(μ-CO) 1.821, Fe2–C(carbyne) 1.864, Fe2–C(Cp, average) 2.134, Fe1–Fe2 2.535, *Se*–C 1.809, C–N(SeCN) 1.160, C–N(carbyne) 1.299; ***cis*-5a**, Fe1–C(CO) 1.763, Fe1–C(μ-CO) 2.002, Fe1–C(carbyne) 1.847, Fe1–C(Cp, average) 2.108, Fe2–N 1.916, Fe2–C(μ-CO) 1.840, Fe2–C(carbyne) 1.856, Fe2–C(Cp, average) 2.122, Fe1–Fe2 2.484, *Se*–C 1.776, C–N(SeCN) 1.162, C–N(carbyne) 1.295. Selected computed angles (°): ***cis*-4a**, Fe1–C(carbyne)–Fe2 84.3, Fe2–*Se*–C 107.5; ***trans*-4a**, Fe1–C(carbyne)–Fe2 86.3, Fe2–*Se*–C 113.5; *cis*-5a, Fe1–C(carbyne)–Fe2 84.3, Fe2–*N*–C 18.0. Cartesian coordinates are provided in the Supplementary Materials.

With the aim of elucidating structural aspects, DFT calculations were carried out on the products obtained from the reaction of **[1a^NCMe^]^+^** with selenocyanate, taking into account the spectroscopic outcomes. Views of the most stable *cis* and *trans* isomers of **4a** and of the most stable *cis* isomer of **5a** are provided in Figure 1; all structures exhibited an α arrangement of the substituents on the aminocarbyne moiety with respect to the selenium-containing ligand. Selected computed bond lengths are summarized in the caption of Figure 1. The *Se*-coordination of the selenocyanate anion was meaningfully bent, with computed Fe2–*Se*–C angles between 107° and 114°. On the other hand, the alternative *N*-coordination was almost linear, with the computed Fe2–*N*–C angle in ***cis*-5a** around 180°. The C–N distance in selenocyanate was scarcely affected by the coordination mode, while on the other hand, the C–Se bond was elongated by more than 0.03 Å when the bonding to iron occurred with the selenium atom. A comparison of the computed bond lengths between ***cis*-4a** and ***cis*-5a** revealed that the Fe2–C bond lengths were negligibly affected by the selenocyanate coordination mode. In accordance with the experimental outcomes, the ν_CN_ stretching of selenocyanate was predicted at slightly higher wavenumbers for the **4a** isomers (unscaled values 2322 cm^−1^ for ***cis*-4a** and 2316 cm^−1^ for ***trans*-4a**) with respect to **5a** (unscaled value 2314 cm^−1^). From a thermodynamic point of view, ***cis*-4a** was less stable than ***cis*-5a** by about 7.7 kcal mol^−1^, and the Gibbs energy difference between ***trans*-4a** and ***cis*-4a** was about 4.7 kcal mol^−1^ in favor of the *cis* isomer. The relative Gibbs energy values, therefore, indicated that the reaction of **[1a^NCMe^]^+^** with selenocyanate afforded a mixture of kinetic products and that conversion of one product into another could take place as promoted by alumina during chromatography. The preference for *N*- rather than *Se*-coordination was computationally established for the mono iron systems [3].

Complex **6a** displayed an infrared band at 2090 cm^−1^, accounting for iron *N*-coordinated cyanide receiving a significant back-donation [71,72,73]; the carbonyl ligands manifested themselves as two IR bands at 1959 and 1808 cm^−1^, suggesting the *trans* configuration of the Cp ligands [74]. In fact, the *cis* isomer of **6a** was previously synthesized from the room temperature reaction of [**1a-NCMe**] with NBu_4_CN [75], and its IR spectrum in the same conditions (CH_2_Cl_2_ solution) consisted of three absorptions at 2091 (C≡N), 1982 (CO), and 1804 cm^−1^ (CO). The ^1^H NMR spectrum of **6a** revealed the presence of a minor amount of the *cis* isomer (<15%). 

**Figure 2:**
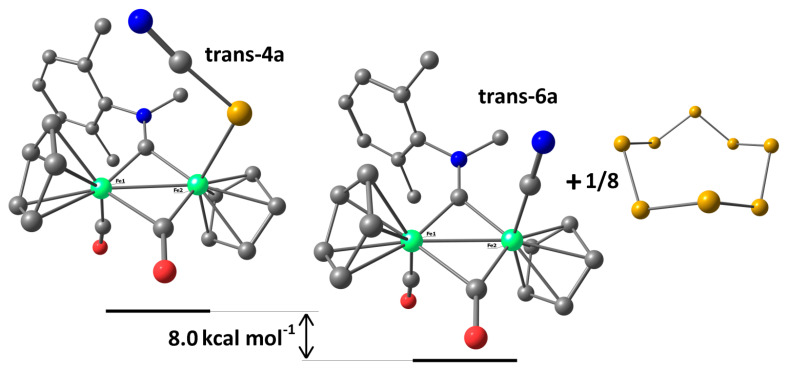
DFT-optimized structures of ***trans*−4a**, ***trans*−6a**, and **Se_8_** (C-PCM/PBEh–3c, acetone as continuous medium). Color map: Fe, green; Se, dark yellow; O, red; N, blue; C, grey; hydrogen atoms omitted for clarity. Selected computed bond lengths (Å): ***trans*−6a**, Fe1–C(CO) 1.760, Fe1–C(μ–CO) 2.089, Fe1–C(carbyne) 1.839, Fe1–C(Cp, average) 2.119, Fe2–C(CN) 1.893, Fe2–C(μ-CO) 1.802, Fe2–C(carbyne) 1.858, Fe2–C(Cp, average) 2.123, Fe1–Fe2 2.523, C–N(CN) 1.162, C–N(carbyne) 1.300; **Se_8_**, Se–Se(average) 2.319. Cartesian coordinates are provided in the Supplementary Materials.

It appeared that the isolation of **6a** from the reaction of **[2a^NCMe^]CF_3_SO_3_** with KSeCN at room temperature was the result of the preliminary coordination of selenocyanate, followed by a rearrangement giving **6a** and releasing one atom of selenium. To confirm this hypothesis, we performed the same reaction in tetrahydrofuran at reflux temperature; in this condition, **6a** was the only isolated product accompanied by the formation of a black solid (presumably elemental selenium). The elimination of selenium probably followed activation of the *Se*–C bond, therefore compounds of type **4a** were most likely involved in the reaction. The computed Gibbs free energy variation for the reaction ***trans*-4a → *trans*-6a** + 1/8 Se_8_ was negative by 8.0 kcal mol^−1^, in alignment with a thermodynamically favorable process. Reactants and products are depicted in Figure 2.

Similarly, the thermal reactions of **[2b,c^NCMe^]CF_3_SO_3_** with KSeCN provided a direct route to the cyanide complexes **6b**–**c** (53–67% yields). These results confirmed the low thermal stability of the selenocyanate complexes with respect to the corresponding cyanide derivatives. The IR spectra of **6b**–**c** were quite similar to that of **6a**, thus suggesting the predominance of *trans* species. The NMR spectra of **6b**–**c** displayed two sets of resonances. These were attributed to *trans* and *cis* isomers in the case of **6b**, with the former largely prevalent (83%), and to α and β isomers in the case of **6c** (isomer ratio 1.3). The diagnostic ^13^C resonance for the cyanide ligand occurred within the range 139.7–141.4 ppm. Former computational studies highlighted the higher stability of the *trans* isomer of **6b** with respect to the corresponding *cis* isomer [76]. 

**Figure 3:**
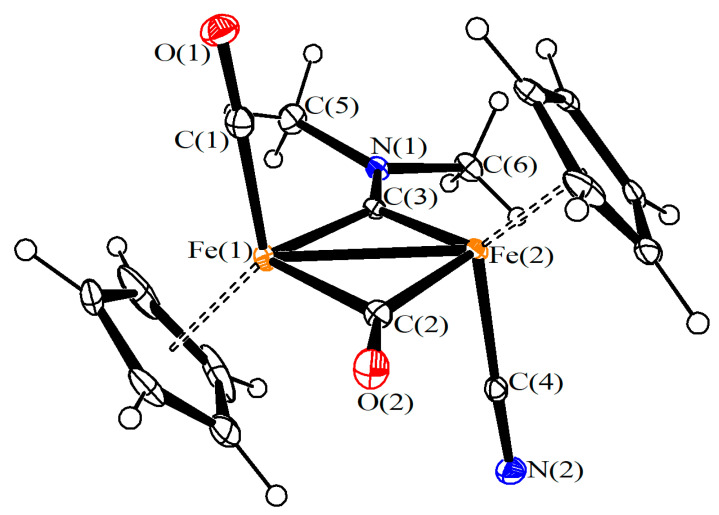
View of the molecular structure (ORTEP drawing) of ***trans*-6b**. Displacement ellipsoids are at the 30% probability level. Selected bond lengths (Å) and angles (°): Fe(1)–Fe(2) 2.5166(4), Fe(1)–C(1) 1.758(2), Fe(1)–C(2) 1.999(2), Fe(2)–C(2) 1.863(2), Fe(1)–C(3) 1.889(2), Fe(2)–C(3) 1.850(2), Fe(2)–C(4) 1.895(2), C(1)–O(1) 1.144(3), C(2)–O(2) 1.173(3), C(3)–N(1) 1.299(3), C(4)–N(2) 1.149(3), N(1)–C(5) 1.473(3), N(1)–C(6) 1.480(3), Fe(1)–C(1)–O(1) 178.4(2), Fe(1)–C(2)–Fe(2) 81.25(9), Fe(1)–C(3)–Fe(2) 84.61(9), Fe(2)–C(4)–N(2) 177.3(2), C(3)–N(1)–C(5) 123.7(2), C(3)–N(1)–C(6) 122.91(19), C(5)–N(1)–C(6) 113.33(18).

The X-ray structure of ***trans*-6b** was determined by single crystal X-ray diffraction (Figure 3). This represented a very rare case of the [Fe_2_Cp_2_(L)(CO)(μ-CO){μ-CN(Me)(R)}]^n^ (L = mono-anionic ligand, n = 0; L = neutral ligand, n = 1+) complex possessing a *trans* geometry. The overall structure and bonding parameters were very similar to those reported for [Fe_2_Cp_2_(NCS)(CO)(μ-CO){μ-CN(Me)_2_}] [39]. The Fe(2)–C(4) interaction [1.895(2) Å] was longer than Fe(1)–C(1) [1.758(2) Å] since the terminal cyanide ligand is a poorer π-acceptor than terminal CO. This caused a considerable asymmetry of the bridging μ-CO ligand, with Fe(1)–C(2) [1.999(2) Å] being significantly longer than Fe(2)–C(2) [1.863(2) Å]. This asymmetry was less marked in the bridging μ-CNMe_2_ ligand [Fe(1)–C(3) = 1.889(2) Å; Fe(2)–C(3) = 1.850(2) Å]. A similar trend was previously observed in *cis*-[Fe_2_(C_5_H_4_Me)_2_(CN)(CO)(μ-CO){μ-CN(Me)_2_}] [77].

## 3. Experimental

### 3.1. Materials and Methods

Reactants and solvents were purchased from Alfa Aesar, Merck, Strem, or TCI Chemicals and were of the highest purity available. Diiron complexes [**1a**–**d**]CF_3_SO_3_ were prepared according to the literature [78,79]. Reactions were conducted under N_2_ atmosphere using standard Schlenk techniques. Products were stored in air once isolated. Dichloromethane and tetrahydrofuran were dried using the solvent purification system mBraun MB SPS5, while acetonitrile was distilled from CaH_2_. IR spectra of solutions were recorded using a CaF_2_ liquid transmission cell (2300–1500 cm^−1^) on a Perkin Elmer Spectrum 100 FTIR spectrometer. IR spectra were processed with Spectragryph software [80]. ^1^H, ^13^C, and ^77^Se NMR spectra were recorded at 298 K on a Jeol JNM-ECZ500R instrument equipped with a Royal HFX Broadband probe. Chemical shifts (expressed in parts per million) were referenced to the residual solvent peak in ^1^H and ^13^C NMR spectra [81] and to an external standard (Me_2_Se) in ^77^Se NMR spectra. NMR spectra were assigned with the assistance of ^1^H–^13^C (*gs*-HSQC and *gs*-HMBC) correlation experiments [82]. NMR signals due to secondary isomeric forms (where it is possible to detect them) are italicized. Elemental analyses were performed using a Vario MICRO cube instrument (Elementar). 

**Cyanate complexes [Fe_2_Cp_2_(k*N*-NCO)(CO)(μ-CO){μ-CN(Me)(R)}] (R = Xyl, 2a; Me, 2b; Cy, 2c).**

### 3.2. General Procedure

A solution of **[1a**–**c]CF_3_SO_3_** (*ca* 0.4 mmol) in MeCN (20 mL) was treated with Me_3_NO∙2H_2_O (1.1 eq.) and the resulting mixture was stirred for 1 h, during which progressive color darkening was observed. The conversion of the starting material into the acetonitrile adduct [**1^NCMe^**]^+^ was checked by IR spectroscopy, as is routine for this type of reaction [75]. Volatiles were removed under vacuum to give a brown residue, which was dissolved in deaerated acetone (30 mL), and NaOCN (3.0 eq.) was added to this solution. The resulting mixture was stirred for 18 h at room temperature and then the solvent was evaporated under reduced pressure. The resulting solid was dissolved in the minimum volume of CH_2_Cl_2_ and this solution was charged on an alumina column. Impurities were separated using neat CH_2_Cl_2_ and neat THF as eluents, and then a brown fraction corresponding to **2a**–**c** was collected with MeCN. The solvent was removed under reduced pressure, and the residue was suspended in hexane for 24 h. After filtration, the separated solid was dried under vacuum.

**Figure 4:**
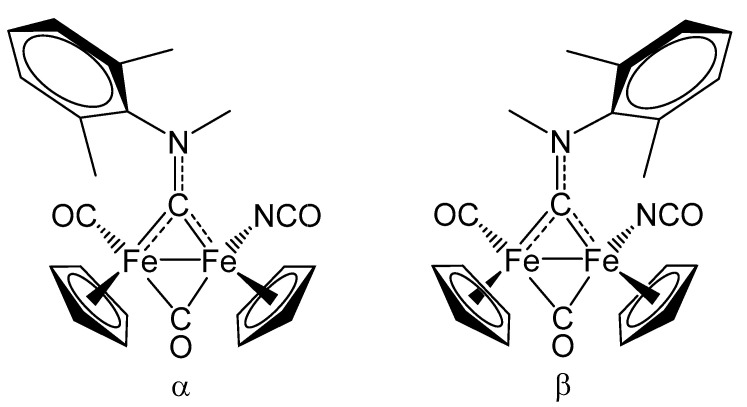
Structure of [Fe_2_Cp_2_(k*N*-NCO)(CO)(μ-CO){μ-CN(Me)(Xyl)}], **2a**.

**[Fe_2_Cp_2_(k*N*-NCO)(CO)(μ-CO){μ-CN(Me)(Xyl)}], 2a** (Figure 4).

From **[1a]CF_3_SO_3_** (268 mg, 0.432 mmol), Me_3_NO∙2H_2_O (53 mg, 0.47 mmol), and NaOCN (187 mg, 1.30 mmol). Dark brown solid, yield 157 mg (75%). Anal. calcd. for C_23_H_22_N_2_O_3_Fe_2_: C, 56.83; H, 4.56; N, 5.76. Found: C, 56.66; H, 4.62; N, 5.68. IR (CH_2_Cl_2_): ῦ/cm^−1^ = 2242 w-br (NCO), 1986 vs. (CO), 1818 s (μ-CO). ^1^H NMR (CDCl_3_): δ/ppm = 7.40–7.28 (m, 3 H, C_6_H_3_); *5.09*, 4.97, 4.45, *4.29* (s, 10 H, Cp); 4.78, *4.47* (s, 3 H, NMe); 0.66, 2.10, *2.06*, *1.91* s, 6 H, C_6_H_3_Me_2_). ^13^C{^1^H} NMR (CDCl_3_): δ/ppm = 338.2 (μ-CN); 264.6 (μ-CO); 211.5 (CO); 148.4 (C-*ipso*); 133.0, 132.8, 130.4, 129.2, 129.1 (C_6_H_3_); 129.8 (NCO); 88.2, 88.1 (Cp); 53.6 (NMe); 19.2, 18.1 (C_6_H_3_Me_2_). Isomer ratio (α/β) = 4.5. 

**Figure 5:**
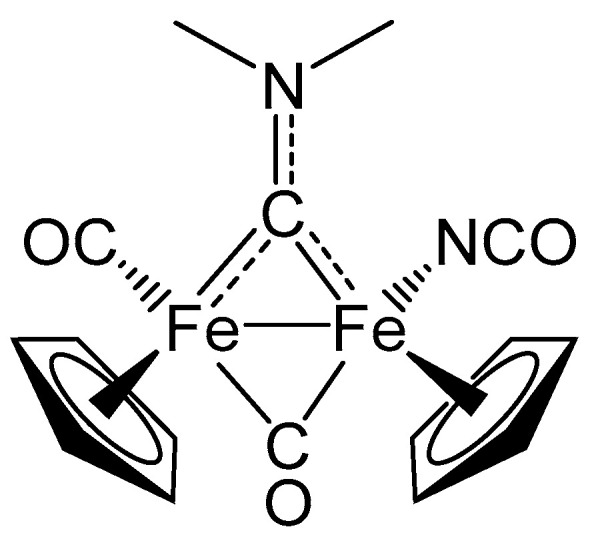
Structure of [Fe_2_Cp_2_(k*N*-NCO)(CO)(μ-CO){μ-CN(Me)(Me)}], **2b** [39].

**[Fe_2_Cp_2_(k*N*-NCO)(CO)(μ-CO){μ-CN(Me)(Me)}], 2b** (Figure 5) [39].

From **[1b]CF_3_SO_3_** (109 mg, 0.205 mmol), Me_3_NO∙2H_2_O (27 mg, 0.25 mmol), and NaOCN (40 mg, 0.61 mmol). Brown solid, yield 66 mg (81%). Anal. calcd. for C_16_H_16_N_2_O_3_Fe_2_: C, 48.53; H, 4.07; N, 7.07. Found: C, 48.72; H, 4.15; N, 7.00. IR (CH_2_Cl_2_): ῦ/cm^−1^ = 2238 vs. (NCO), 1981 vs. (CO), 1804 s (μ-CO), 1578 w (μ-CN). ^1^H NMR (CDCl_3_): δ/ppm = 4.74, 4.62 (s, 10 H, Cp); 4.6 *, 4.21 (s, 6 H, NMe_2_); * Hidden by Cp resonance.

**Figure 6:**
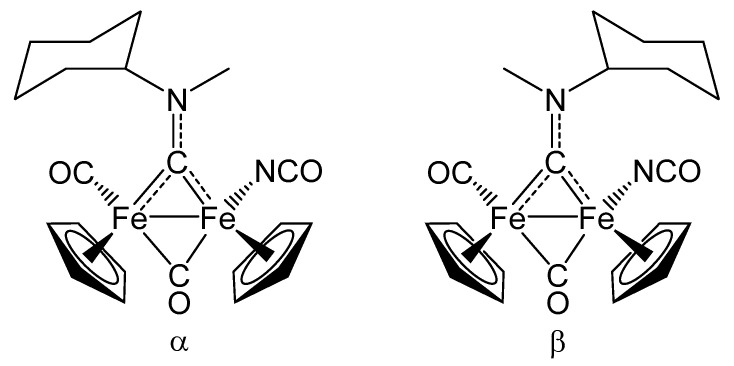
Structure of [Fe_2_Cp_2_(k*N*-NCO)(CO)(μ-CO){μ-CN(Me)(Cy)}], **2c**.

**[Fe_2_Cp_2_(k*N*-NCO)(CO)(μ-CO){μ-CN(Me)(Cy)}], 2c** (Figure 6).

From **[1c]CF_3_SO_3_** (256 mg, 0.427 mmol), Me_3_NO∙2H_2_O (52 mg, 0.47 mmol), and NaOCN (184 mg, 1.28 mmol). Dark brown solid, yield 133 mg (67%). Anal. calcd. for C_21_H_24_N_2_O_3_Fe_2_: C, 54.35; H, 5.21; N, 6.04. Found: C, 54.29; H, 5.30; N, 6.08. IR (CH_2_Cl_2_): ῦ/cm^−1^ = 2255 m (NCO); 1984 vs. (CO), 1818 s (μ-CO), 1540 w (μ-CN). ^1^H NMR (CDCl_3_): δ/ppm = 5.59, 4.8 * (m, 1 H, CH^Cy^); *4.99*, 4.90, 4.87, *4.81* (s, 10 H, Cp); 4.44, *4.08* (s, 3 H, NMe); 2.71–2.46, 2.22–2.11, 1.82–1.49, 1.39–1.21 (m, 10 H, CH_2_^Cy^). * Partially hidden by Cp resonances. ^13^C{^1^H} NMR (CDCl_3_): δ/ppm = *329.3*, 328.9 (μ-CN); *266.4*, 266.1 (μ-CO); 211.6, *210.9* (CO); 130.3, *130.0* (NCO); *88.6*, 88.1, 87.2, *86.9* (Cp); 78.1 (CH^Cy^); *45.9*, 45.6 (NMe); 32.5, 31.3, 30.9, 28.6, 26.0, 25.9, 25.7, 25.2, 25.1, 24.4 (CH_2_^Cy^). Isomer ratio (α/β) = 1.3. 

**Figure 7:**
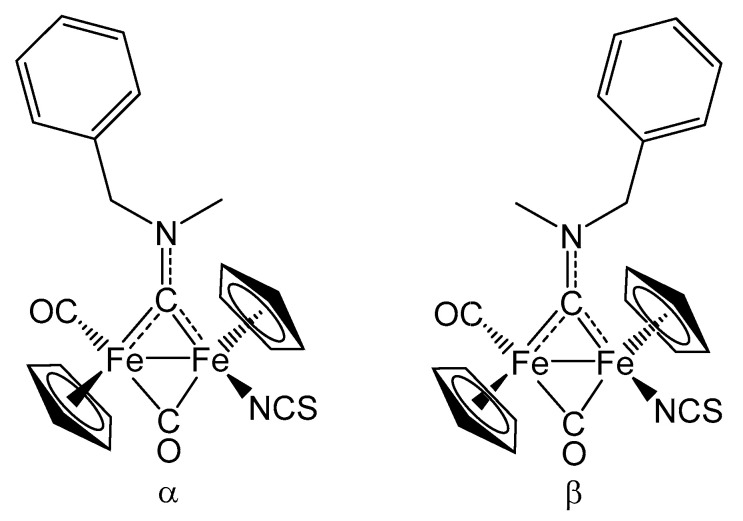
Thiocyanate complex [Fe_2_Cp_2_(k*N*-NCS)(CO)(μ-CO){μ-CN(Me)(CH_2_Ph)}], **3**.

**Thiocyanate complex [Fe_2_Cp_2_(k*N*-NCS)(CO)(μ-CO){μ-CN(Me)(CH_2_Ph)}], 3** (Figure 7).

A mixture of [**1d^NCMe^]CF_3_SO_3_**, freshly generated from [**1d]CF_3_SO_3_** (90 mg, 0.148 mmol) according to the procedure above, and NBu_4_SCN (220 mg, 0.733 mmol) in CH_2_Cl_2_ (12 mL) was stirred for 3 h at room temperature. The final solution was directly charged on an alumina column, and elution with neat dichloromethane afforded the fraction corresponding to the title product. Thus, volatiles were removed under vacuum to give an orange solid. Yield 58 mg (80%). Anal. calcd. for C_22_H_20_Fe_2_N_2_O_2_S: C, 54.13; H, 4.13; N, 5.74; S, 6.57. Found: C, 54.32; H, 4.04; N, 5.62; S, 6.49. IR (CH_2_Cl_2_): ῦ/cm^−1^ = 2114 s (NCS); 1970 vs. (CO), 1810 s (μ-CO), 1535 w (μ-CN). ^1^H NMR (CDCl_3_): δ/ppm = 7.59–7.39 (m, 5 H, CH_2_Ph); 6.90, *6.17*, 5.87, *5.50* (d, ^2^J_HH_ = 14 Hz, 2 H, CH_2_Ph); *4.92*, 4.83, 4.61, *4.56* (s, 10 H, Cp); *4.47*, 4.06 (s, 3 H, NMe). ^13^C{^1^H} NMR (CDCl_3_): δ/ppm = *341.5*, 340.6 (μ-CN); 264.1, *263.2* (μ-CO); *212.3*, 211.6 (CO); 141.2, *140.6* (NCS); 134.2–127.1 (CH_2_Ph); *89.5*, 89.4, 88.2 (Cp); *71.0*, 70.5 (CH_2_Ph); 49.8, *49.0* (NMe). Isomer ratio (α/β) = 2. 

**Reactivity of [1a-NCMe]CF_3_SO_3_ with KSeCN: isolation of 4a, 5a and 6a.**

The acetonitrile adduct **[1a^NCMe^]CF_3_SO_3_** was prepared from **[1a]CF_3_SO_3_** (176 mg, 0.283 mmol) and Me_3_NO∙2H_2_O (35 mg, 0.31 mmol), as described above. Then, **[1a^NCMe^]CF_3_SO_3_** was dissolved in deaerated acetone (30 mL), and KSeCN (122 mg, 0.85 mmol) was added. The resulting mixture was stirred for 16 h at room temperature [final IR spectrum (CH_2_Cl_2_): ῦ/cm^−1^ = 2112 m (SeCN), 2068 w, 1970 vs-br (CO), 1812 s-br (μ-CO)]. Volatiles were evaporated under reduced pressure, hence a solution of the residue in the minimum volume of dichloromethane was charged on an alumina column. Neat CH_2_Cl_2_ allowed the separation of a light green fraction corresponding to **4a**, while CH_2_Cl_2_/THF (9/1 *v*/*v*) mixture was used to collect a red fraction corresponding to **5a**. After removing impurities with THF, elution with MeCN/MeOH (9/1 *v*/*v*) led to the separation of a dark green fraction corresponding to **6a**. For each fraction, the solvent was removed under reduced pressure, and the residue was suspended in hexane for 24 h. After filtration, the solid product was dried under vacuum. 

**Figure 8:**
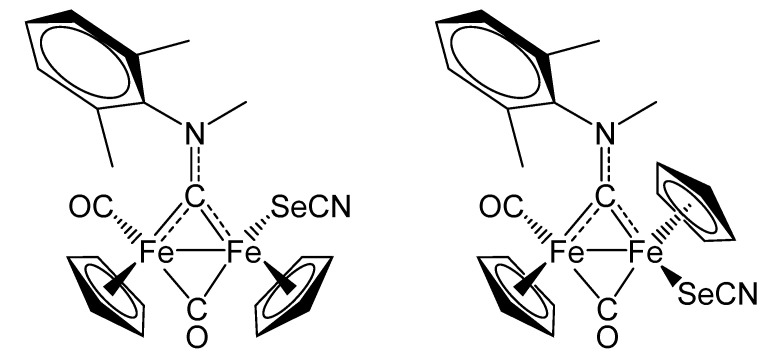
Structure of [Fe_2_Cp_2_(k*Se*-SeCN)(CO)(μ-CO){μ-CN(Me)(Xyl)}], **4a**.

**[Fe_2_Cp_2_(k*Se*-SeCN)(CO)(μ-CO){μ-CN(Me)(Xyl)}], 4a** (Figure 8).

**Figure 9:**
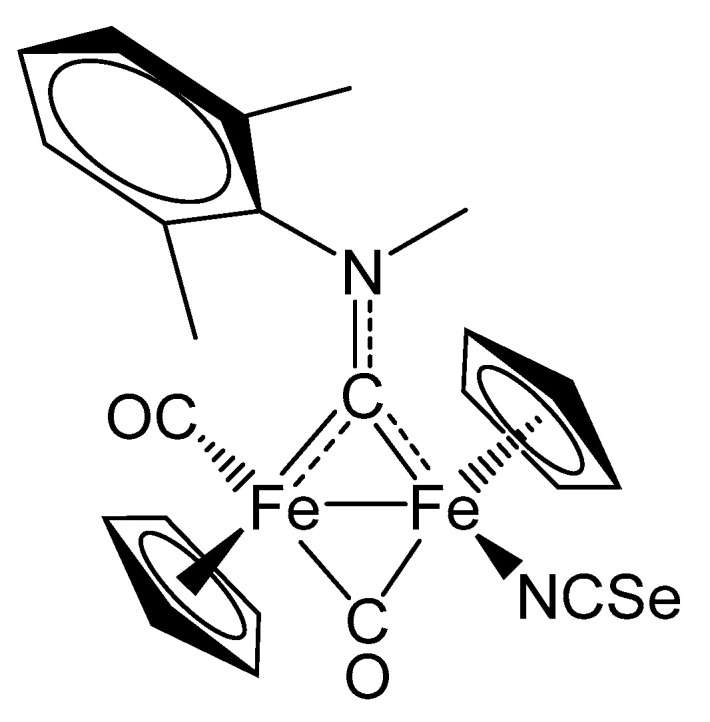
Structure of [Fe_2_Cp_2_(k*N*-NCSe)(CO)(μ-CO){μ-CN(Me)(Xyl)}], **5a**.

**[Fe2Cp2(k*N*-NCSe)(CO)(μ-CO){μ-CN(Me)(Xyl)}], 5a** (Figure 9).

Light brown solid, yield 23 mg (15%). Anal. calcd. for C_23_H_22_N_2_O_2_Fe_2_Se: C, 50.31; H, 4.04; N, 5.10. Found: C, 50.15; H, 4.12; N, 5.18. IR (CH_2_Cl_2_): ῦ/cm^−1^ = 2113 m (SeCN), 1970 vs. (CO), 1812 s (μ-CO). ^1^H NMR (CDCl_3_): δ/ppm = 7.42–7.30 (m, 3 H, C_6_H_3_); 5.07, *4.76*, *4.38*, 4.08 (s, 10 H, Cp); *4.92*, 4.53 (s, 3 H, NMe); *2.57*, 2.48, 2.44, *2.39* (s, 6 H, C_6_H_3_Me_2_). ^13^C{^1^H} NMR (CDCl_3_): δ/ppm = *345.4*, 342.1 (μ-CN); 263.1, *262.5* (μ-CO); *212.6*, 211.1 (CO); 149.0, *148.9* (C-*ipso*); 136.1–128.7 (C_6_H_3_ + SeCN); 89.7, *89.2*, 88.1, *87.8* (Cp); 54.7, *53.8* (NMe); *19.0*, 18.9, 18.8, *18.5* (C_6_H_3_Me_2_). ^77^Se{^1^H} NMR (CDCl_3_): δ/ppm = −232.5, *−246.9*. Isomer ratio (*cis/trans*) = 1.7.

**Figure 10:**
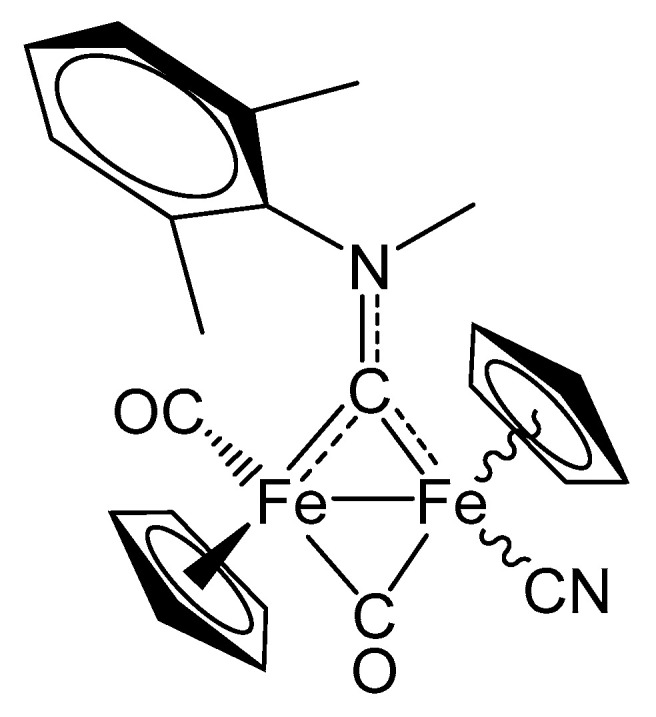
Structure of [Fe_2_Cp_2_(CN)(CO)(μ-CO){μ-CN(Me)(Xyl)}], **6a**.

**[Fe_2_Cp_2_(CN)(CO)(μ-CO){μ-CN(Me)(Xyl)}], 6a** (Figure 10).

Dark red solid, yield 20 mg (13%). Anal. calcd. for C_23_H_22_N_2_O_2_Fe_2_Se: C, 50.31; H, 4.04; N, 5.10. Found: C, 50.46; H, 4.12; N, 5.22. IR (CH_2_Cl_2_): ῦ/cm^−1^ = 2109 m (NCSe), 1960 vs. (CO), 1804 s (μ-CO). ^1^H NMR (CDCl_3_): δ/ppm = 7.35–7.27 (m, 3 H, C_6_H_3_); 4.85, 4.27 (s, 10 H, Cp); 4.83 (s, 3 H, NMe); 2.54, 2.42 (s, 6 H, C_6_H_3_Me_2_). ^13^C{^1^H} NMR (CDCl_3_): δ/ppm = 340.4 (μ-CN); 263.3 (μ-CO); 213.1 (CO); 149.2 (C-*ipso*); 134.2, 132.6, 129.6, 129.3, 128.6 (C_6_H_3_); 108.4 (NCSe); 89.3, 88.9 (Cp); 53.4 (NMe); 18.5, 18.4 (C_6_H_3_Me_2_). ^77^Se{^1^H} NMR (CDCl_3_): δ/ppm = −340.2.

Green solid, yield 64 mg (48%). Anal. calcd. for C_23_H_22_N_2_O_2_Fe_2_: C, 58.76; H, 4.72; N, 5.96. Found: C, 58.46; H, 4.90; N, 5.79. IR (CH_2_Cl_2_): ῦ/cm^−1^ = 2090 w (C≡N), 1959 vs. (CO), 1808 s (μ-CO). ^1^H NMR (CDCl_3_): δ/ppm = 7.3–7.2 (m, 3 H, C_6_H_3_); 4.85, 4.8 *, *4.35*, 4.29 (s, 10 H, Cp); 4.52, *4.45* (s, 3 H, NMe); 2.64, 2.52, 2.40, 2.23 (s, 6 H, C_6_H_3_Me_2_). * Partially hidden by Cp resonance of major isomer. ^13^C{^1^H} NMR (CDCl_3_): δ/ppm = 340.3, *337.0* (μ-CN); *261.6*, 260.8 (μ-CO); 213.0, *211.5* (CO); 148.9, *148.5* (C-*ipso*); 140.2, *139.7* (C≡N); *134.4*, 134.3, *132.9*, 132.7, *130.4*, 129.5, 129.3, *128.7*, 128.5, *128.3* (C_6_H_3_); 89.5, 88.8, *87.3*, *86.5* (Cp); 53.6, *52.9* (NMe); 18.7, *18.6*, 18.5, *17.9* (C_6_H_3_Me_2_). Isomer ratio (*trans/cis*) = 6. When the reaction of **[1a^NCMe^]CF_3_SO_3_** (179 mg, 0.28 mmol) with KSeCN (122 mg, 0.85 mmol) was conducted in THF at reflux temperature, complete conversion of the starting material into **6a** was checked via IR spectroscopy after 4 h; **6a** (^1^H NMR) was isolated in 45% yield after alumina chromatography. 

**Reactivity of other acetonitrile complexes with KSeCN: evidence for the formation of selenocyanate derivatives and isolation of [Fe_2_Cp_2_(CN)(CO)(μ-CO){μ-CN(Me)(R)}] (R = Me, 6b; R= Cy, 6c).**

### 3.3. General Procedure

The acetonitrile adduct was prepared from **[1b,c]CF_3_SO_3_** as described above, then it was dissolved in deaerated acetone (30 mL), and KSeCN (3.0 eq.) was added to this solution. The resulting mixture was stirred for 16 h at room temperature. The volatiles were evaporated under reduced pressure, thus the residue was dissolved in dichloromethane and this solution was charged on an alumina column. Neat CH_2_Cl_2_ allowed the separation of impurities, and then CH_2_Cl_2_/THF (2/1 *v*/*v*) mixture was used to collect a brown fraction. Elution with MeCN/MeOH (9/1 *v*/*v*) led to the separation of a dark green fraction corresponding to **6b**–**c**. For each fraction, the solvent was removed under reduced pressure, and the residue was suspended in hexane for 24 h. After filtration, isolated products (i.e., brown solid and green solid) were dried under vacuum.

(1) From **[1b]CF_3_SO_3_** (226 mg, 0.426 mmol), Me_3_NO∙2H_2_O (52 mg, 0.47 mmol), and KSeCN (184 mg, 1.28 mmol). Brown solid (yield 52%), ῦ/cm^−1^ = 2110 m (SeCN), 1966 vs. (CO), 1804 s (μ-CO). **6b**, yield 23%.

When the reaction of **[1b^NCMe^]CF_3_SO_3_** (102 mg, 0.19 mmol) with KSeCN (122 mg, 0.85 mmol) was conducted in THF (or acetone) at reflux temperature, complete conversion of the starting material into **6b** was checked via IR spectroscopy after 4 h; **6b** was isolated in 53% yield after alumina chromatography. 

**Figure 11:**
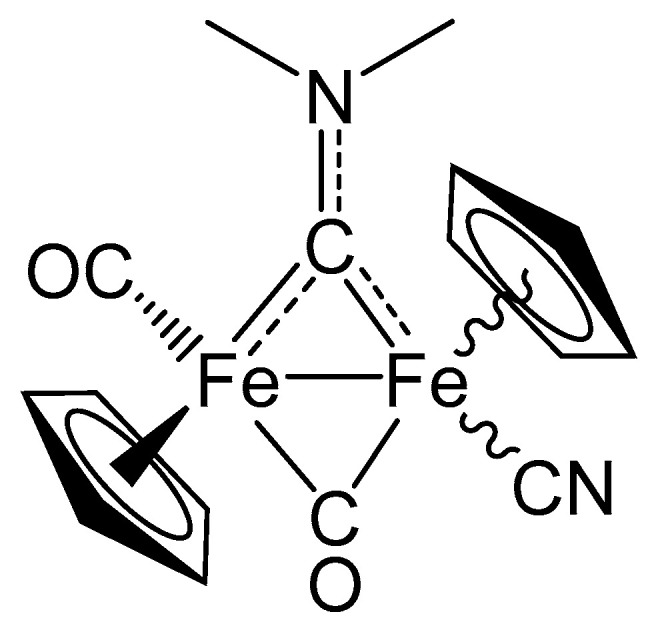
Structure of [Fe_2_Cp_2_(CN)(CO)(μ-CO){μ-CNMe_2_}], **6b**.

**[Fe_2_Cp_2_(CN)(CO)(μ-CO){μ-CNMe_2_}], 6b** (Figure 11).

Green solid. Anal. calcd. for C_16_H_16_N_2_O_2_Fe_2_: C, 50.57; H, 4.24 N, 7.37. Found: C, 50.44; H, 4.14; N, 7.28. IR (CH_2_Cl_2_): ῦ/cm^−1^ = 2089 w (C≡N), 1962 vs. (CO), 1805 s (μ-CO), 1568 w (μ-CN). ^1^H NMR (CDCl_3_): δ/ppm = 4.84, *4.80*, *4.76*, 4.69 (s, 10 H, Cp); 4.33, *4.24*, 4.21, *4.10* (s, 6 H, NMe_2_). ^13^C{^1^H} NMR (CDCl_3_): δ/ppm = 337.0 (μ-CN); 261.5 (μ-CO); 212.3, *210.8* (CO); 141.2 (C≡N); 89.9, 89.0, *87.0*, *86.5* (Cp); *52.3*, 51.9 (NMe_2_). Isomer ratio (*trans/cis*) = 5. Crystals of ***trans*-6b** suitable for X-ray analysis were obtained by slow diffusion of hexane layered on an acetone solution of **6b** at −30 °C. 

(2) From **[1c]CF_3_SO_3_** (256 mg, 0.427 mmol), Me_3_NO∙2H_2_O (52 mg, 0.47 mmol), and KSeCN (184 mg, 1.28 mmol). Brown solid (yield 41%), ῦ/cm^−1^ = 2110 w (SeCN); 1964 vs. (CO), 1803 s (μ-CO). **6c**, yield 36%.

When the reaction of **[1c^NCMe^]CF_3_SO_3_** (186 mg, 0.30 mmol) with KSeCN (122 mg, 0.85 mmol) was conducted in THF at reflux temperature, complete conversion of the starting material into **6c** was checked via IR spectroscopy after 4 h; **6c** was isolated in 67% yield after alumina chromatography. 

**Figure 12:**
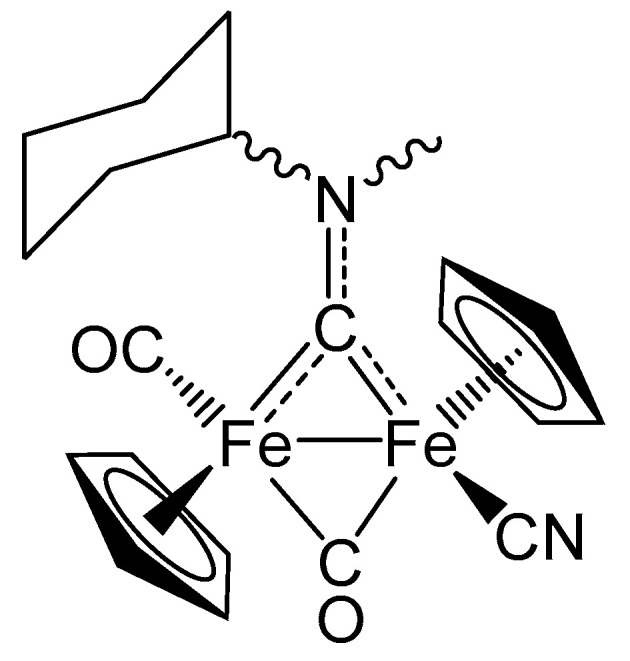
Structure of [Fe_2_Cp_2_(CN)(CO)(μ-CO){μ-CN(Me)(Cy)}], **6c**.

**[Fe_2_Cp_2_(CN)(CO)(μ-CO){μ-CN(Me)(Cy)}], 6c** (Figure 12).

Green solid. Anal. calcd. for C_21_H_24_N_2_O_2_Fe_2_: C, 56.29; H, 5.40; N, 6.25. Found: C, 56.44; H, 5.31; N, 6.38. IR (CH_2_Cl_2_): ῦ/cm^−1^ = 2089 w (C≡N); 1959 vs. (CO), 1803 s (μ-CO); 1528 w (μ-CN). ^1^H NMR (CDCl_3_): δ/ppm = 5.33, *5.13* (m, 1 H, CH^Cy^); 4.80, *4.78*, *4.65*, 4.64 (s, 10 H, Cp); *4.17*, 4.04 (s, 3 H, NMe); 2.27–2.17, 2.10–1.83, 1.64, 1.34–1.24 (m, 10 H, CH_2_^Cy^). ^13^C{^1^H} NMR (CDCl_3_): δ/ppm = 336.1, *335.8* (μ-CN); 262.2, *261.8* (μ-CO); 212.6, *212.3* (CO); *141.4*, 140.6 (C≡N); 90.2, 90.0, *89.2*, *89.0* (Cp); 75.9, *75.3* (CH^Cy^); *44.5*, 44.2 (NMe); 32.0, *31.6*, 31.5, *31.0*, 26.2, 25.8, *25.7*, 25.5, 25.3 (CH_2_^Cy^). Isomeric ratio (α/β) = 1.3. 

## 4. X-ray Crystallography

Crystal data and collection details for ***trans*-6b** are reported in Table 1. Data were recorded on a Bruker APEX II diffractometer equipped with a PHOTON2 detector using Mo–Kα radiation. The structures were solved by direct methods and refined by full-matrix least-squares based on all data using *F*^2^ [83]. Hydrogen atoms were fixed at calculated positions and refined using a riding model.

## 5. Computational Details

Geometry optimizations were performed using the PBEh-3c method, which is a reparametrized version of PBE0 [84] (with 42% HF exchange) that uses a split-valence double-zeta basis set (def2-mSVP) [85,86] and adds three corrections considering dispersion, basis set superposition, and other basis set incompleteness effects [87,88,89]. The C-PCM implicit solvation model was added to PBEh-3c calculations, considering acetone as a continuous medium [90,91]. IR simulations were carried out using harmonic approximation, from which zero-point vibrational energies and thermal corrections (T = 298.15 K) were obtained [92]. The software used was ORCA version 5.0.3 [93].

## 6. Conclusions

New diiron aminocarbyne complexes with a terminal chalcogen-containing pseudohalide ligand were synthesized, and their stereochemistry and thermodynamic stability were investigated by IR and NMR spectroscopy and DFT calculations. Combined with previous findings, this work highlights that *N*-coordination generally prevails over the alternative coordination mode, although different kinetic products may be formed, and that the reactivity of the NCE^−^ ligand increases along the sequence O (inertness) < S (electrophilic addition) < Se (chalcogen elimination). In particular, we provide clear evidence for the formation of diiron cyanide complexes from the fragmentation of the selenocyanate fragment.

## Figures and Tables

**Table 1:** Crystal data and measurement details for ***trans*-6b**.

	*trans*-6b
Formula	C_16_H_16_Fe_2_N_2_O_2_
FW	380.01
T, K	100(2)
*λ*, Å	0.71073
Crystal system	Monoclinic
Space group	P*2_1_*/c
*a*, Å	8.7226(2)
*b*, Å	12.4479(3)
*c*, Å	14.1827(4)
*β*, °	107.2940(10)
Cell Volume, Å^3^	1470.31(6)
Z	4
*D_c_*, g∙cm^−3^	1.717
μ, mm^−1^	1.980
F(000)	776
Crystal size, mm	0.16 × 0.15 × 0.13
θ limits, °	2.222–26.996
Reflections collected	23,142
Independent reflections	3205 [R_in_ = 0.0474]
Data/restraints/parameters	3205/0/201
Goodness on fit on F^2^	1.074
*R*_1_ (*I* > 2σ(*I*))	0.0283
*wR*_2_ (all data)	0.0677
Largest diff. peak and hole, e Å^−3^	0.562/−0.444

## Data Availability

Not applicable.

## Supplementary Materials

The following supporting information can be downloaded at: https://www.mdpi.com/article/10.3390/molecules28073251/s1, NMR spectra of products and DFT data. CCDC reference number 2225698 (*trans*-6b) contains the supplementary crystallographic data for the X-ray study reported in this paper.
