# Supplementary material for: Diiron Aminocarbyne Complexes with NCE− Ligands (E = O, S, Se)
Source: Molecules. 2023 Apr 5;28(7):3251. doi: 10.3390/molecules28073251 (PMC10096932; doi:10.3390/molecules28073251)
Supplement: Supplementary file 1 [file molecules-28-03251-s001.zip › Supporting Information Revised.pdf]

Diiron Aminocarbyne Complexes with NCE^{-} Ligands (E = O, S, Se)

Giulio Bresciani, Stefano Zacchini, Guido Pampaloni, Marco Bortoluzzi, Fabio Marchetti

Supporting Information

Table of contents *Pages*

Figures S1-S19: ^{1}H, ^{13}C and ^{77}Se NMR spectra of products S2-S12

Cartesian coordinates of the DFT-optimized structures S13-S17

Figure S1. ^{1}H NMR spectrum (401 MHz, $CDCl_3$) of **2a**

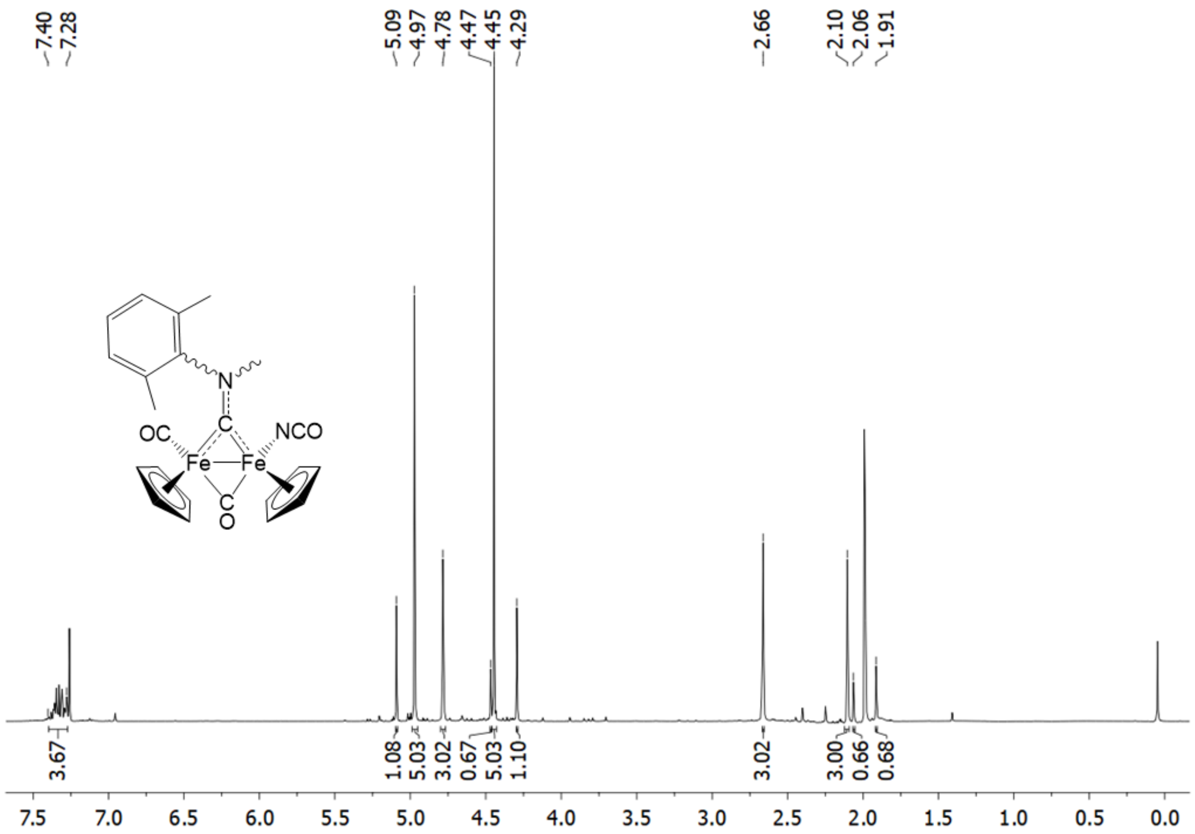

Figure S2. $^{13}C\{^{1}H\}$ NMR spectrum (101 MHz, $CDCl_3$) of **2a**

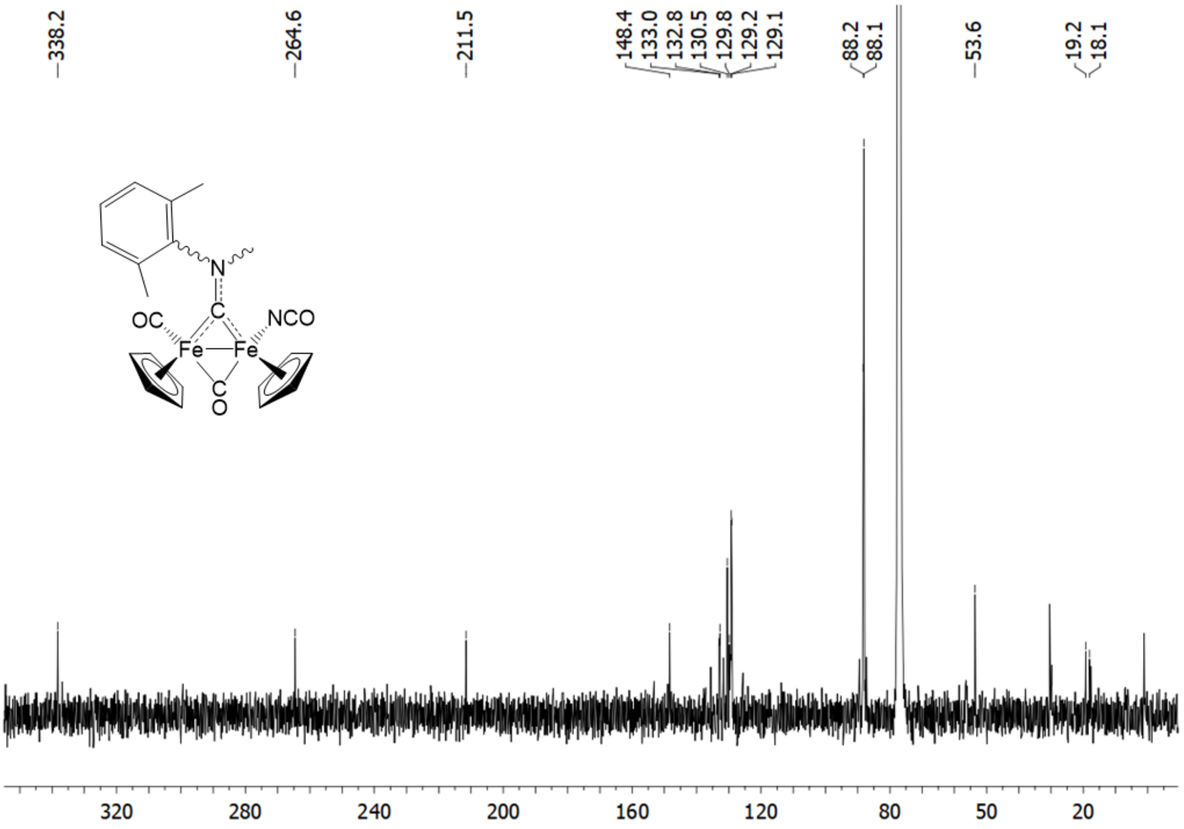

Figure S3. ^{1}H NMR spectrum (401 MHz, $CDCl_3$) of **2b**

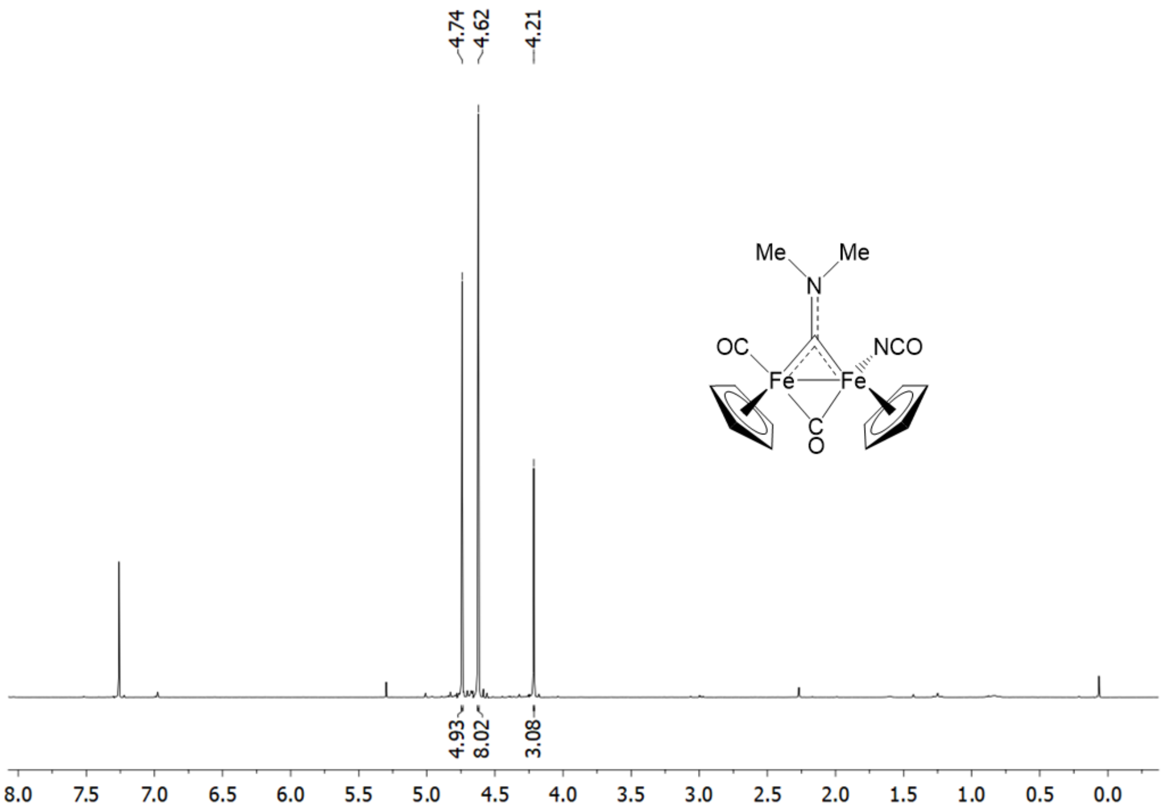

Figure S4. ^{1}H NMR spectrum (401 MHz, $CDCl_3$) of **2c**

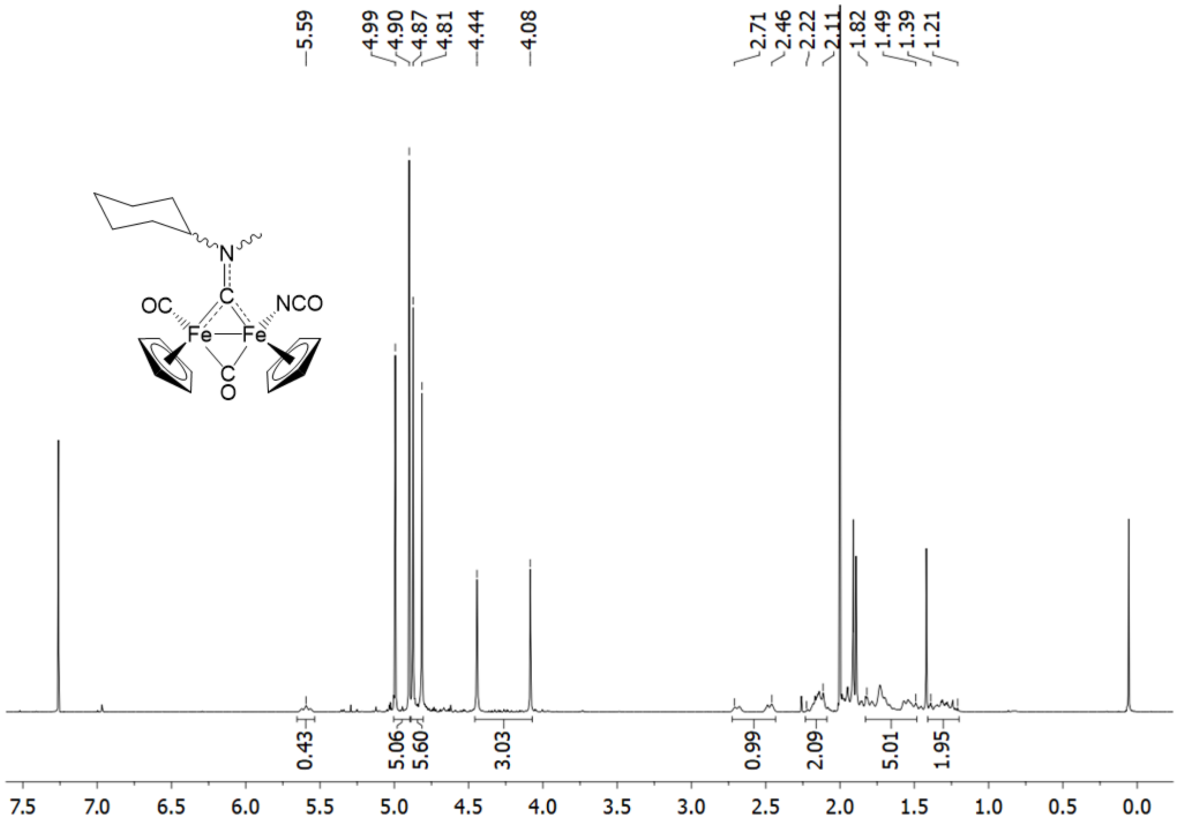

Figure S5. ^{13}C{^{1}H} NMR spectrum (101 MHz, $CDCl_3$) of **2c**

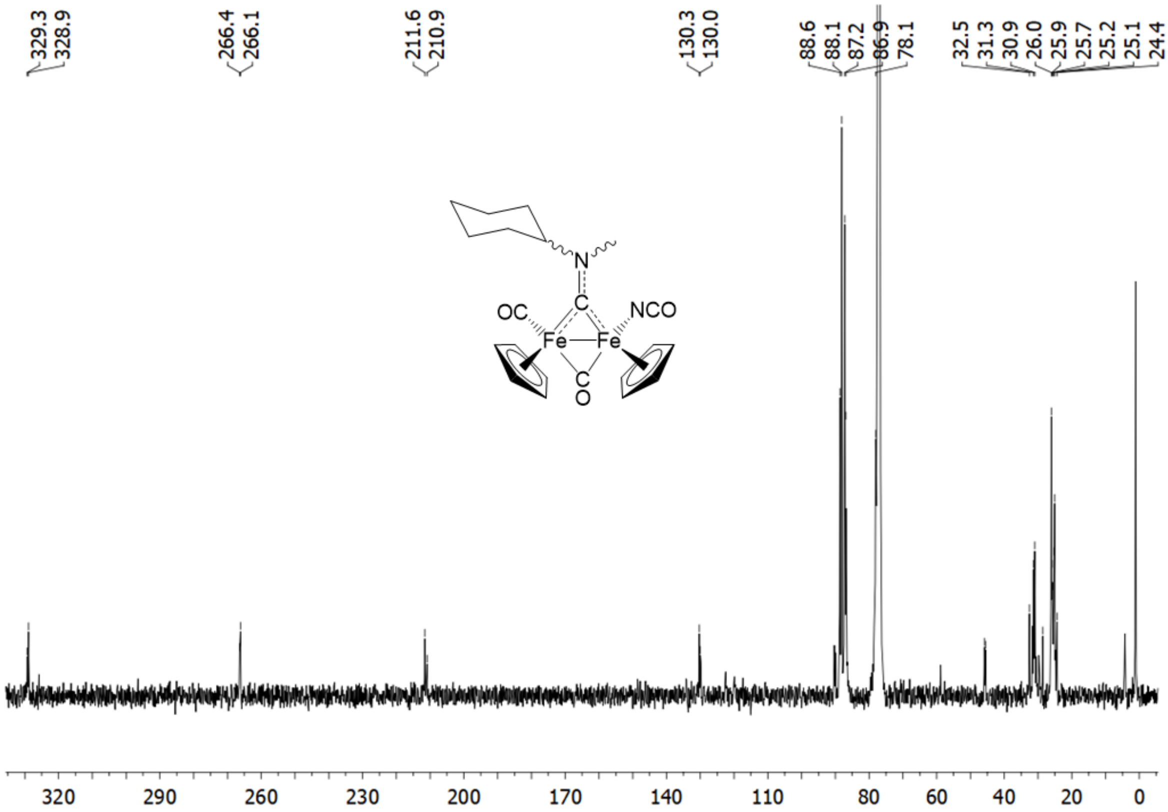

Figure S6. ^{1}H NMR spectrum (401 MHz, $CDCl_3$) of **3**

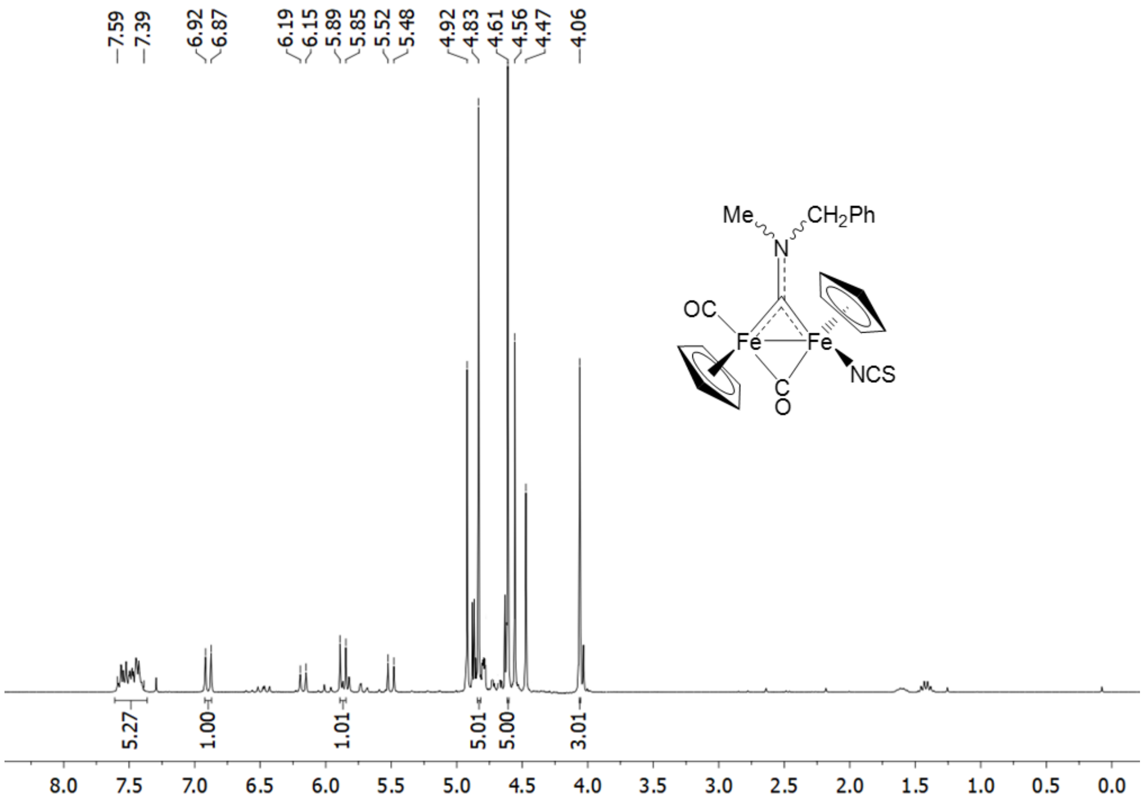

Figure S7. $^{13}C\{^{1}H\}$ NMR spectrum (101 MHz, $CDCl_3$) of **3**

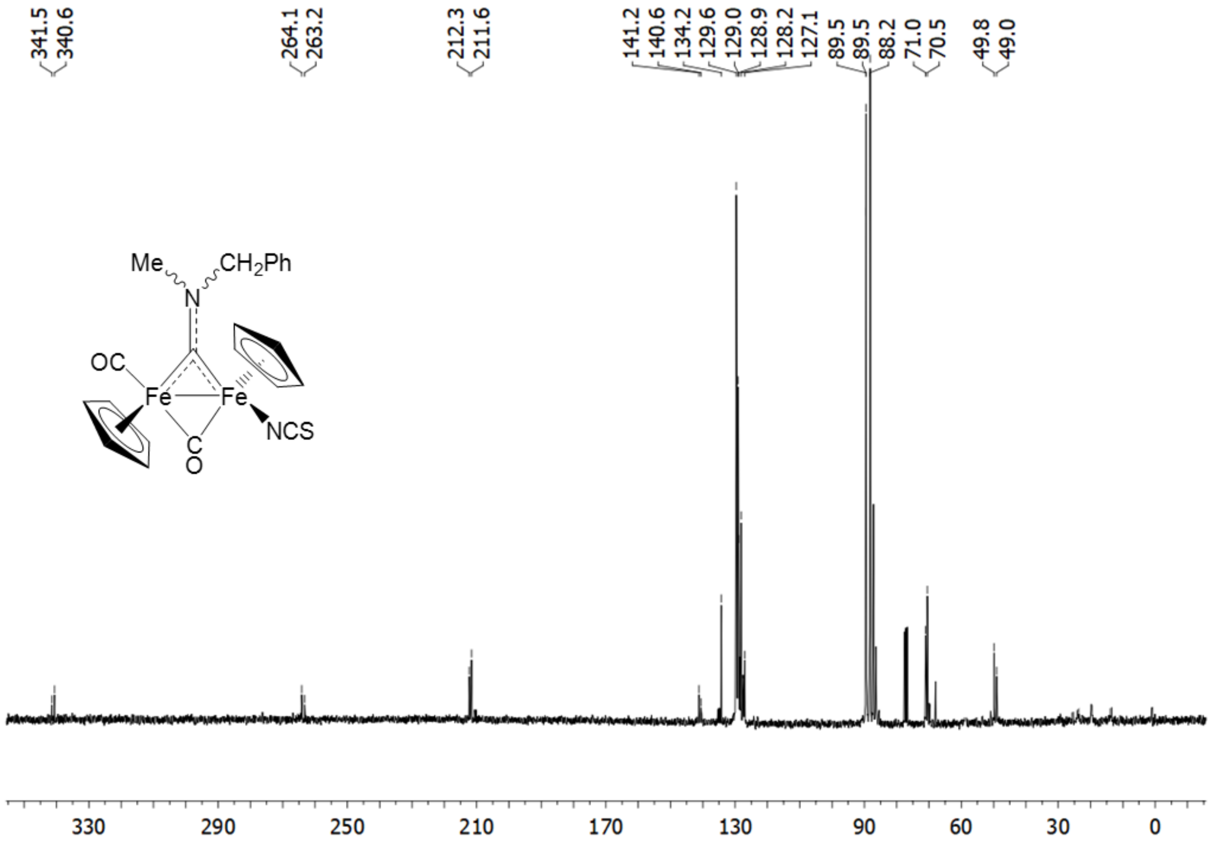

Figure S8. ^{1}H NMR spectrum (401 MHz, $CDCl_3$) of **4a**

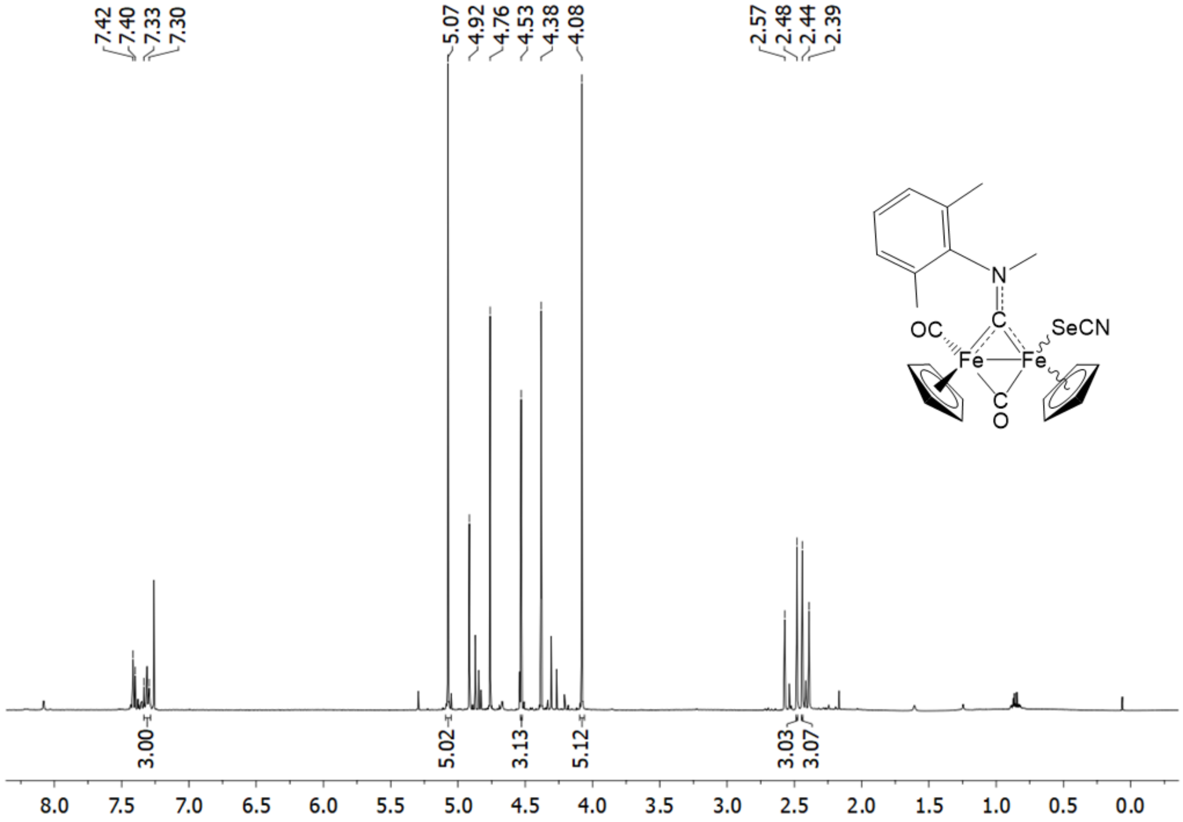

Figure S9. $^{13}C\{^{1}H\}$ NMR spectrum (101 MHz, $CDCl_3$) of **4a**

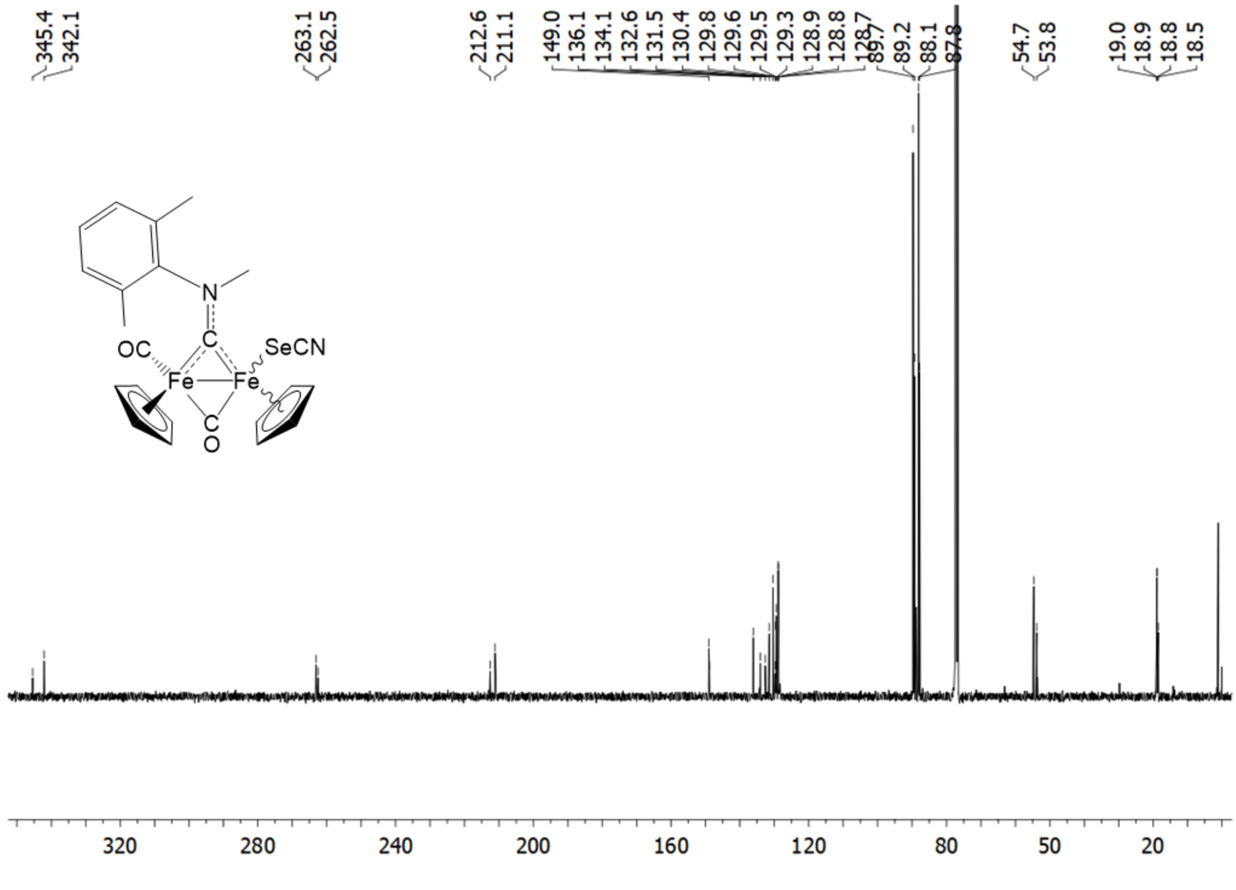

Figure S10. $^{77}Se\{^1H\}$ NMR spectrum (76 MHz, $CDCl_3$) of **4a**

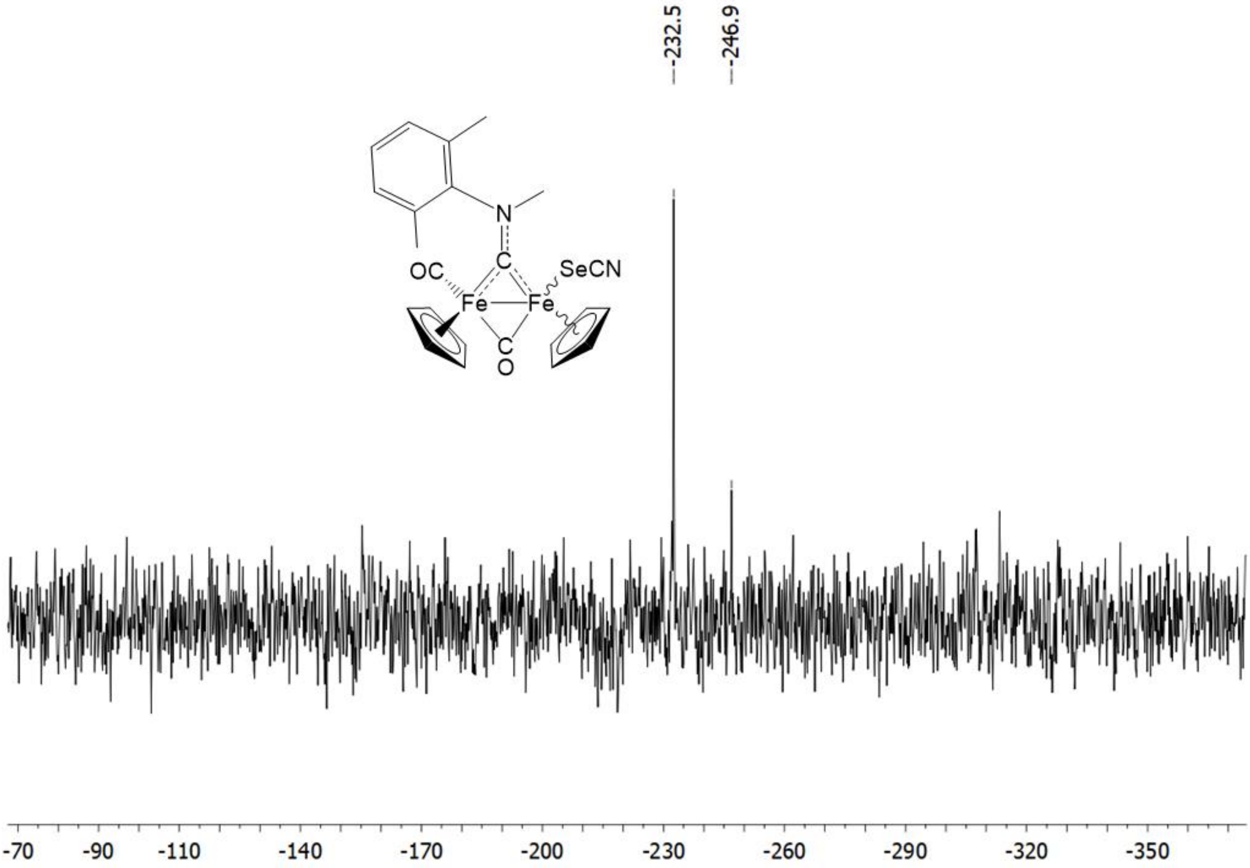

Figure S11. ^{1}H NMR spectrum (401 MHz, $CDCl_3$) of **5a**

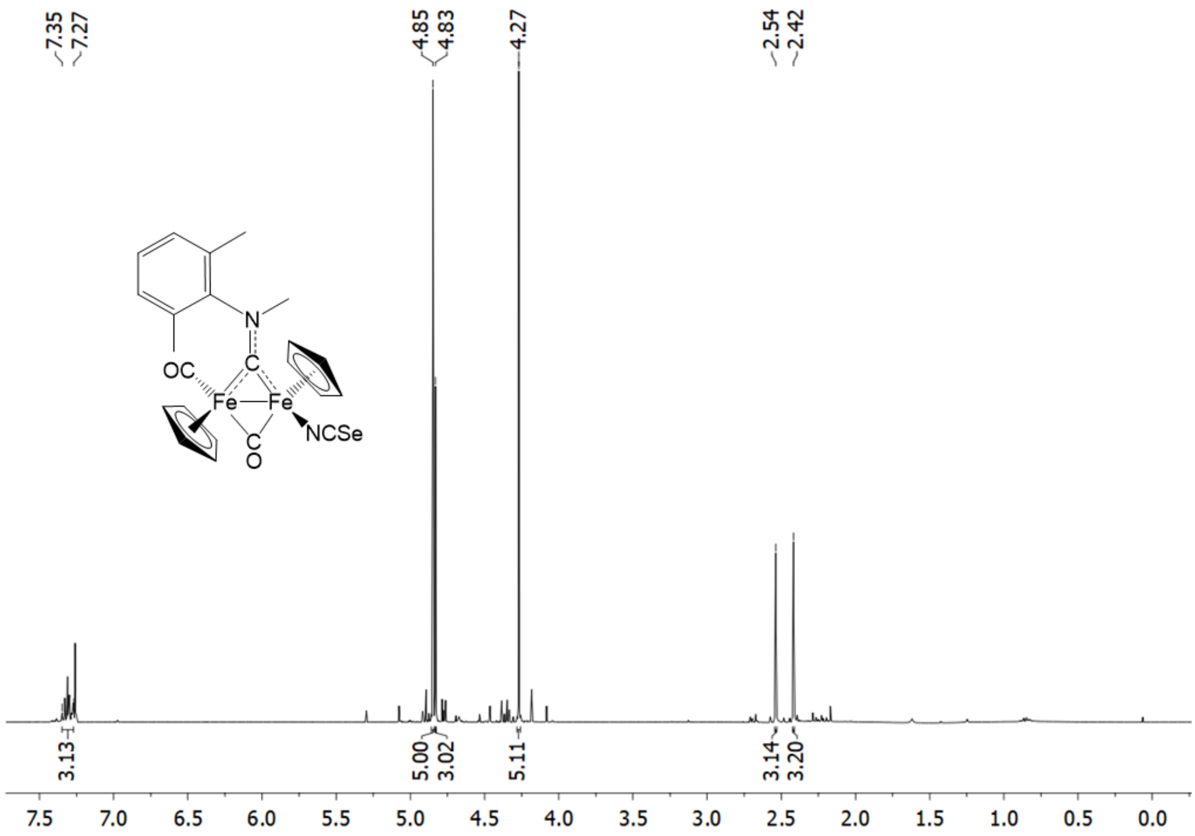

Figure S12. $^{13}C\{^{1}H\}$ NMR spectrum (101 MHz, $CDCl_3$) of **5a**

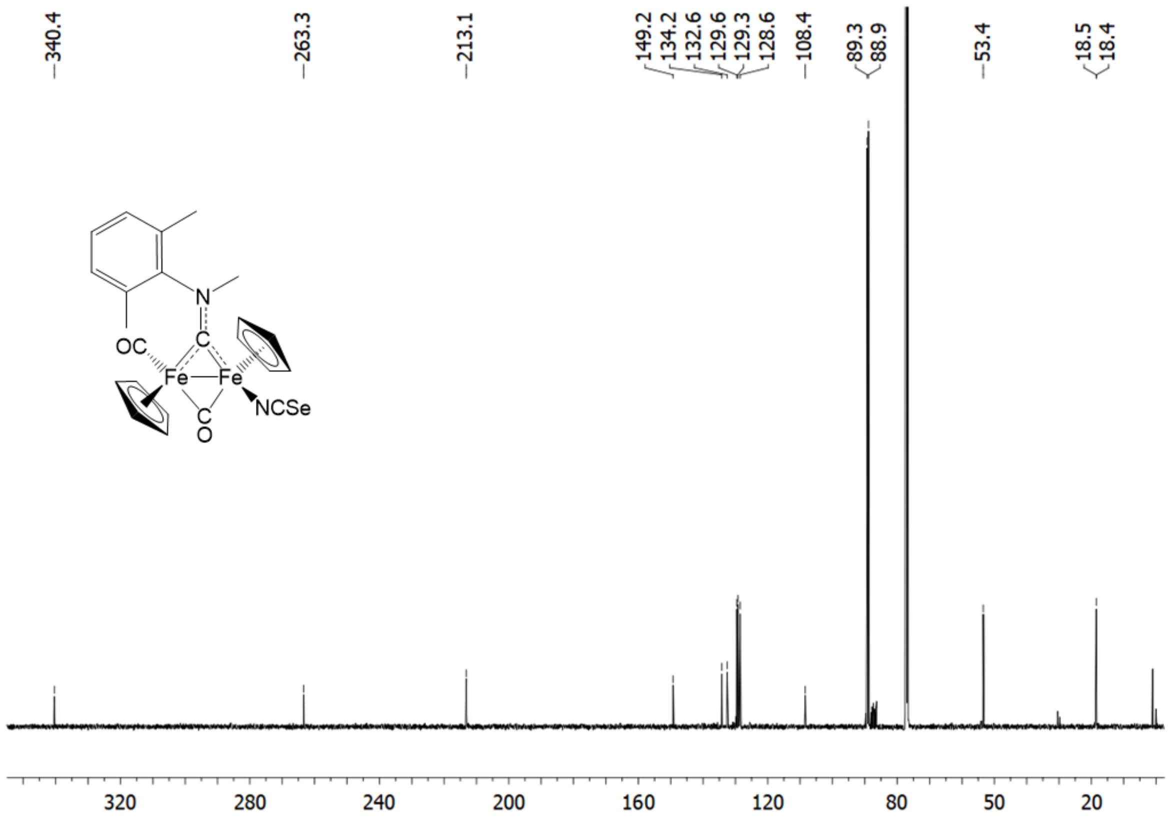

Figure S13. $^{77}Se\{^1H\}$ NMR spectrum (76 MHz, $CDCl_3$) of **5**

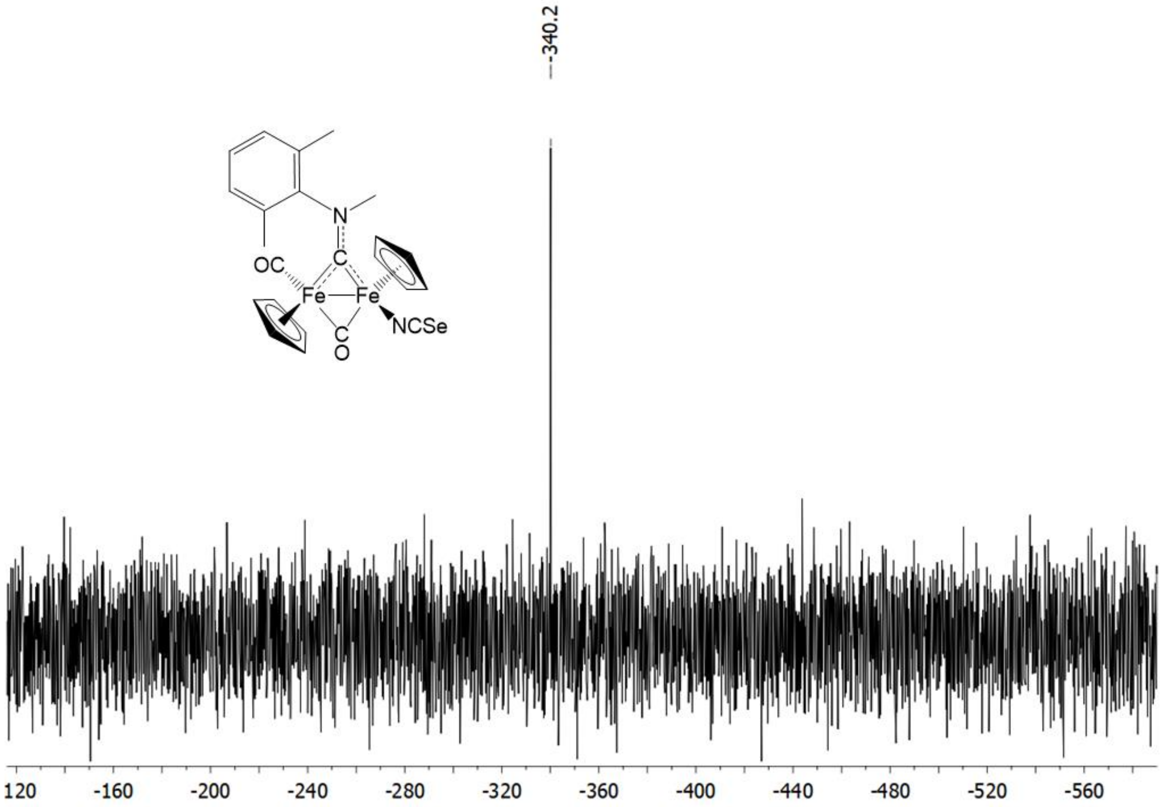

Figure S14. ^{1}H NMR spectrum (401 MHz, $CDCl_3$) of **6a**

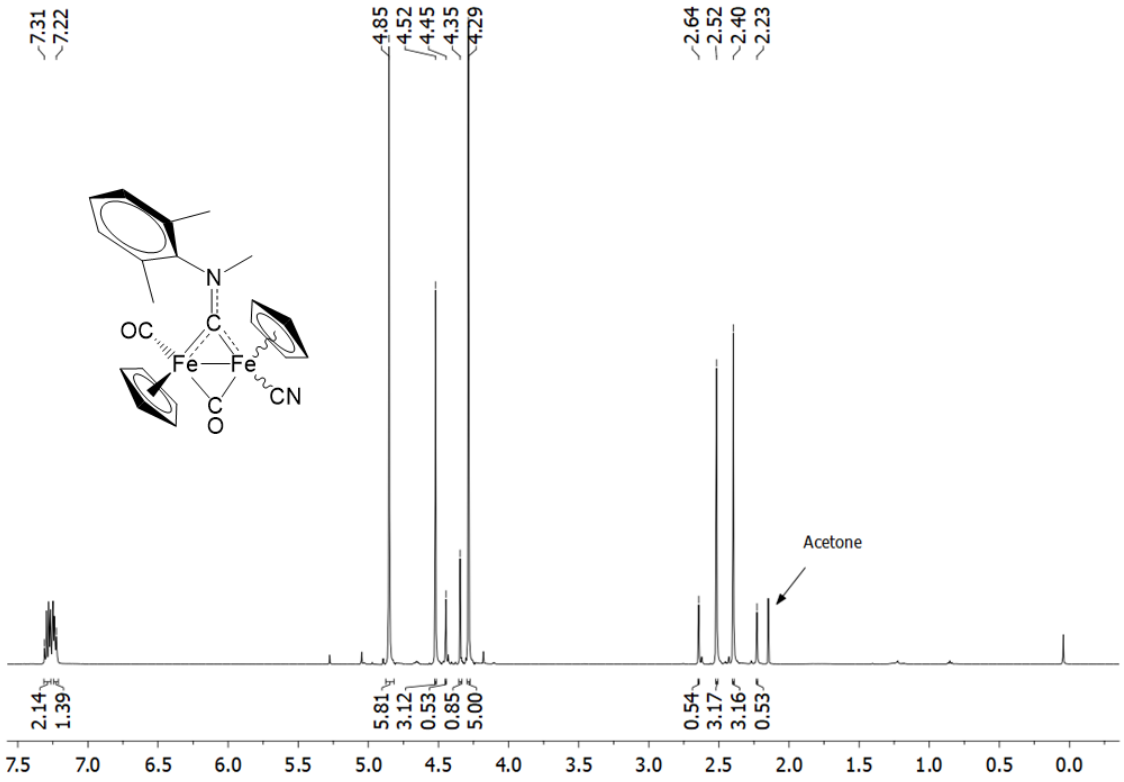

Figure S15. $^{13}C\{^{1}H\}$ NMR spectrum (101 MHz, $CDCl_3$) of **6a**

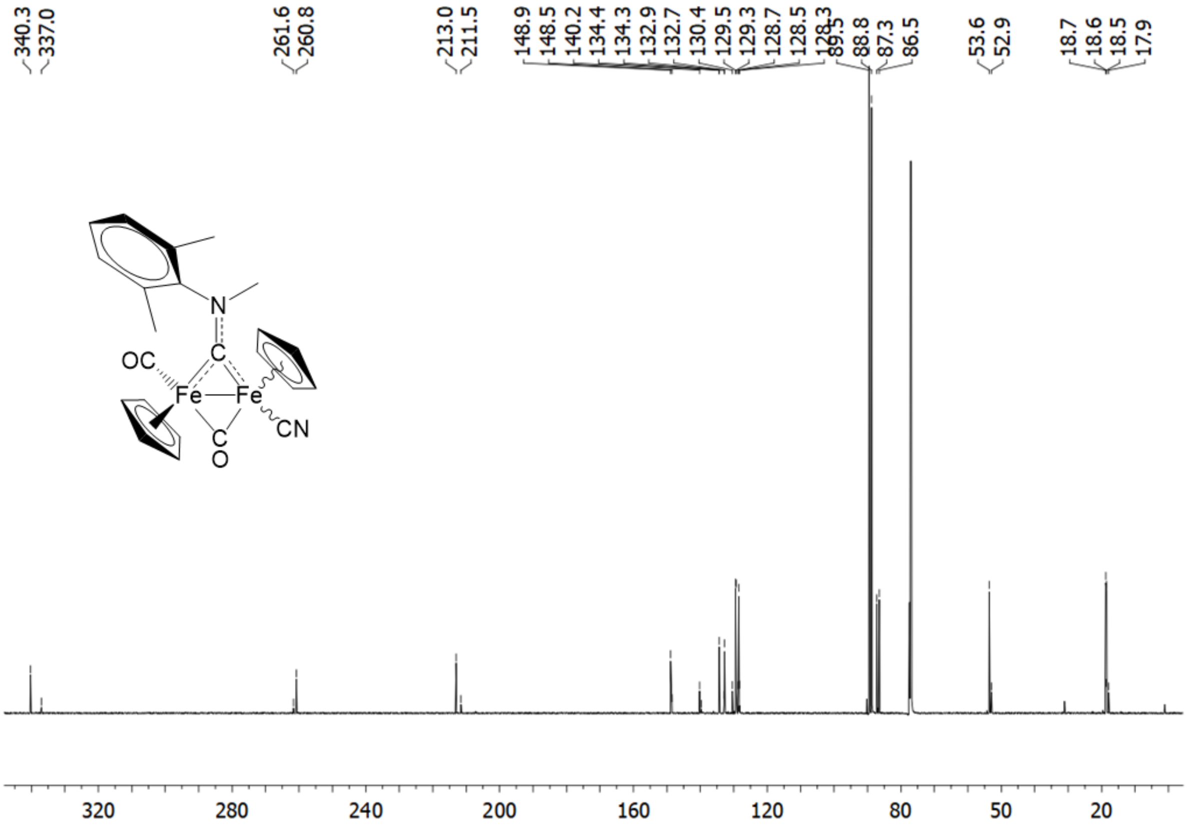

Figure S16. ^{1}H NMR spectrum (401 MHz, $CDCl_3$) of **6b**

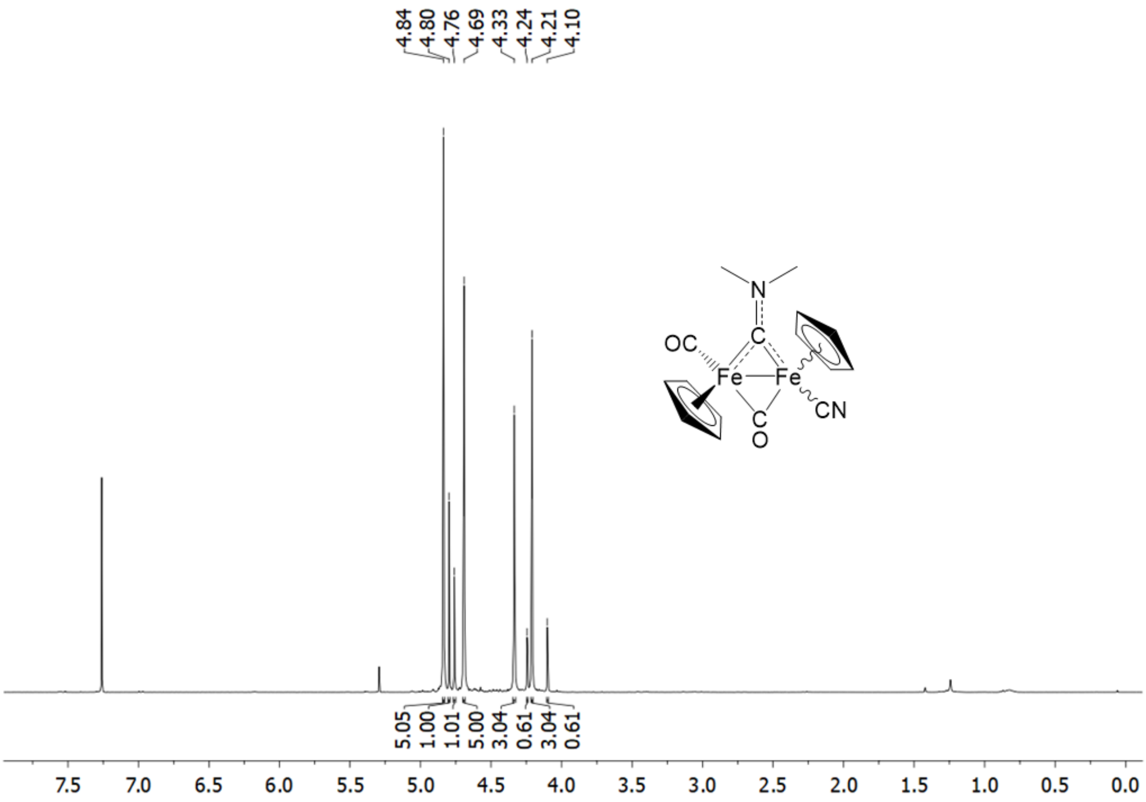

Figure S17. ^{13}C{^{1}H} NMR spectrum (101 MHz, $CDCl_3$) of **6b**

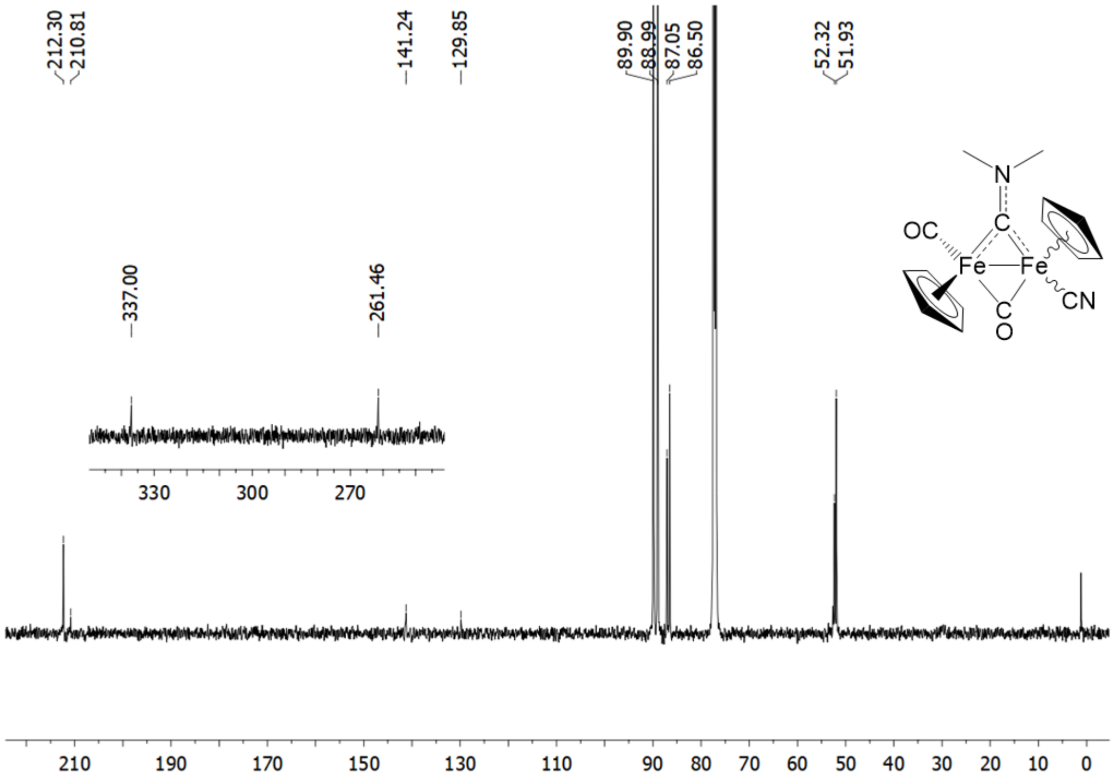

Figure S18. ^{1}H NMR spectrum (401 MHz, $CDCl_3$) of **6c**

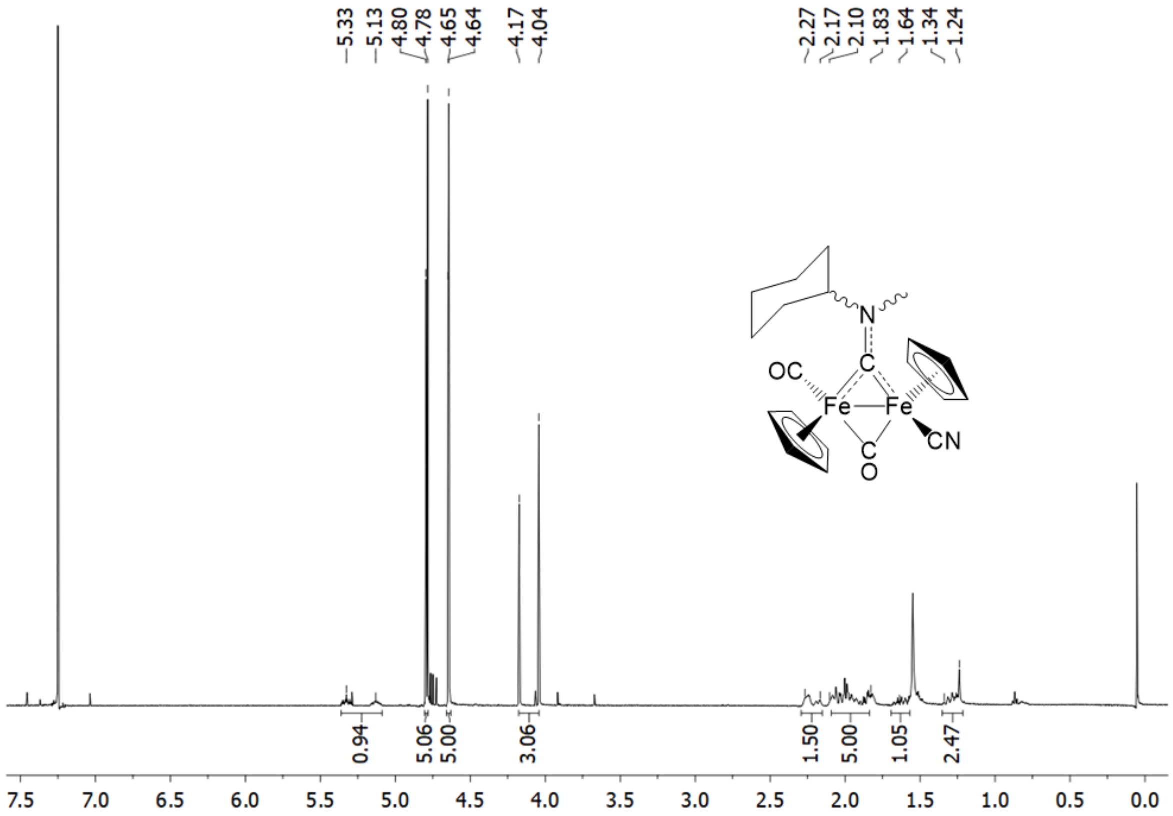

Figure S19. ^{13}C{^{1}H} NMR spectrum (101 MHz, $CDCl_3$) of **6c**.

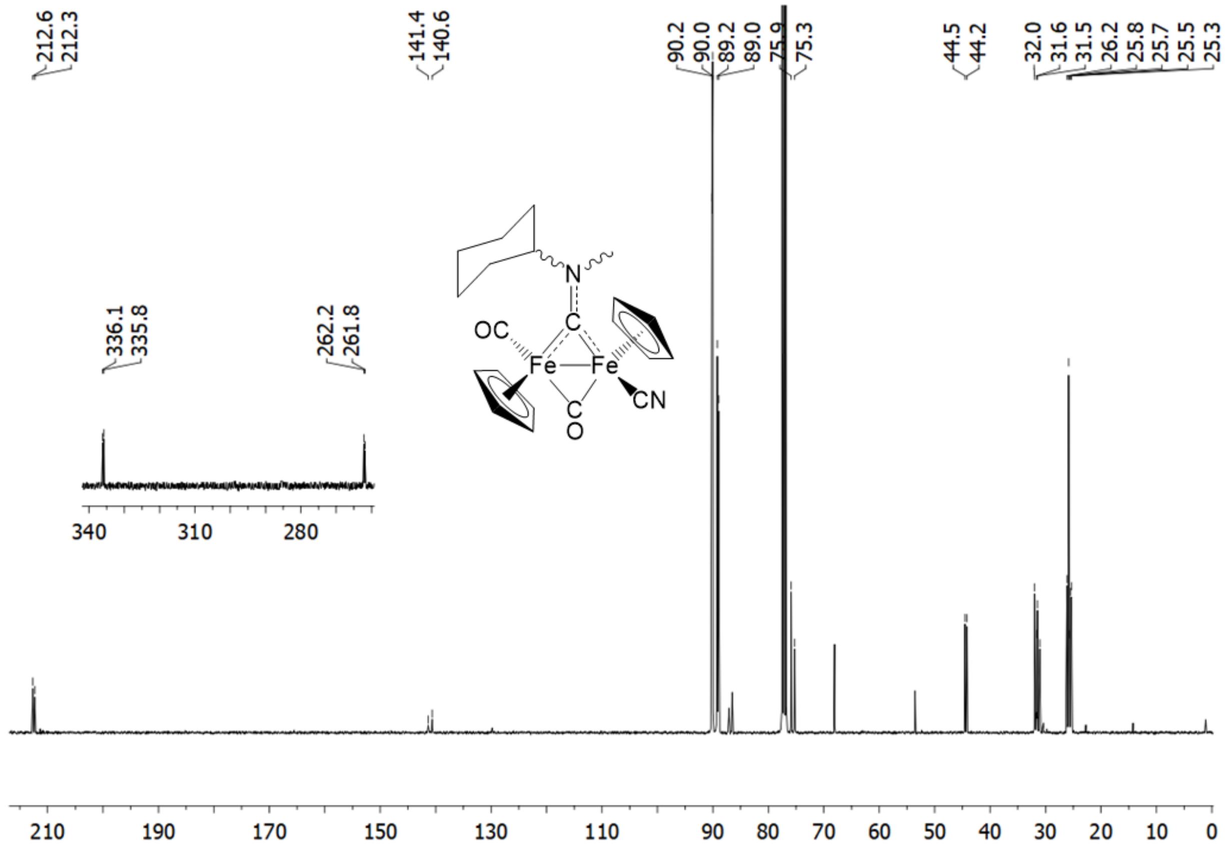

Cartesian coordinates of the DFT-optimized structures

cis-4a

52
Fe2C23N2H22SeO2

C	0.488666000	-0.295300000	0.547976000
Fe	1.984800000	0.529969000	1.228091000
Fe	-0.400392000	1.094501000	1.667050000
O	0.084534000	-1.113510000	-0.172239000
C	1.154248000	1.418867000	2.627909000
N	1.506390000	2.049108000	3.706170000
C	2.881029000	2.165671000	4.192833000
H	3.537528000	1.513219000	3.632707000
H	2.919246000	1.884645000	5.243743000
H	3.222556000	3.196386000	4.094465000
H	3.997734000	-0.951361000	-0.172798000
C	3.638064000	0.052608000	-0.002249000
C	4.039169000	0.914674000	1.056553000
H	2.186318000	0.344955000	-1.655697000
H	4.774618000	0.685095000	1.814200000
C	3.337884000	2.131105000	0.913102000
H	3.430643000	2.999673000	1.548545000
C	2.475652000	2.012744000	-0.200576000
H	1.824307000	2.783713000	-0.582398000
C	2.683968000	0.731978000	-0.778196000
Se	2.221148000	-1.560899000	2.463776000
C	3.109837000	-1.189275000	3.999839000
N	3.686267000	-1.019687000	4.991555000
C	-0.895116000	-0.175114000	2.789869000
O	-1.335779000	-0.958855000	3.487760000
H	0.042019000	3.821224000	0.822865000
C	-0.700790000	3.041209000	0.889548000
C	-1.579213000	2.808398000	1.979259000
H	-0.488844000	2.001781000	-1.065787000
H	-1.637352000	3.376477000	2.893927000
C	-2.407170000	1.716367000	1.635330000
H	-3.191883000	1.302550000	2.253752000
C	-2.044042000	1.262415000	0.342540000
H	-2.499797000	0.446546000	-0.198104000
C	-0.992645000	2.091089000	-0.113842000
C	0.556353000	2.757619000	4.521885000
C	-1.284980000	4.126878000	6.051492000
C	-0.107442000	2.077129000	5.543238000
C	0.374754000	4.123952000	4.297711000
C	-0.563166000	4.795308000	5.075908000
C	-1.049737000	2.782808000	6.287649000
H	-0.732657000	5.851288000	4.903631000
H	-1.595624000	2.269348000	7.070139000
H	-2.023392000	4.658535000	6.637604000
C	1.160717000	4.887898000	3.272334000
H	1.544200000	4.258877000	2.472146000
H	2.018421000	5.379854000	3.736124000
H	0.549009000	5.667769000	2.820583000
C	0.206822000	0.657853000	5.916172000
H	0.703244000	0.098394000	5.128100000
H	-0.696646000	0.119467000	6.199388000
H	0.871217000	0.643626000	6.782904000

trans-4a

52
Fe2C23N2H22SeO2

C	0.752829000	-0.373137000	0.767889000
Fe	2.006398000	0.855054000	1.253919000
Fe	-0.320759000	0.633626000	2.234111000
O	0.445139000	-1.249600000	0.079906000
C	1.188282000	1.535455000	2.784571000
N	1.551858000	2.308031000	3.764091000
C	2.924368000	2.784351000	3.949817000
H	3.626800000	2.038996000	3.591506000
H	3.107272000	2.956793000	5.007830000
H	3.078478000	3.725313000	3.420799000
H	0.072650000	-0.434281000	4.899277000
C	-0.620803000	-0.381230000	4.077008000
C	-0.708594000	-1.314638000	3.026358000
H	-1.837496000	1.444082000	4.519083000
H	-0.080479000	-2.184308000	2.904831000
C	-1.772552000	-0.925982000	2.169027000
H	-2.106033000	-1.444383000	1.282729000
C	-2.344965000	0.243816000	2.711602000
H	-3.186258000	0.784771000	2.300001000
C	-1.623055000	0.603852000	3.875954000
H	4.386536000	0.175015000	-0.250508000
C	3.635264000	0.942528000	-0.121824000
C	3.700774000	2.033765000	0.787608000
H	2.107711000	0.367072000	-1.622874000
H	4.516393000	2.247715000	1.461349000
C	2.537842000	2.813476000	0.602753000
H	2.299297000	3.730861000	1.120644000
C	1.744777000	2.194948000	-0.388552000
H	0.812645000	2.566279000	-0.784009000
C	2.437872000	1.041622000	-0.846852000
C	-0.928029000	1.963668000	1.254382000
O	-1.426504000	2.801499000	0.663034000
Se	3.372682000	-0.833727000	2.385032000
C	2.551130000	-1.590914000	3.807736000
N	2.102944000	-2.132366000	4.730704000
C	0.603879000	2.794694000	4.732219000
C	-1.273701000	3.645075000	6.570905000
C	-0.221015000	3.872669000	4.405951000
C	0.562501000	2.187113000	5.990346000
C	-0.399752000	2.620615000	6.896927000
C	-1.171721000	4.273372000	5.341392000
H	-0.460866000	2.145618000	7.868583000
H	-1.834984000	5.095506000	5.099831000
H	-2.024534000	3.964187000	7.282220000
C	1.538540000	1.125006000	6.406709000
H	1.968181000	0.584734000	5.566979000
H	1.064155000	0.398910000	7.066211000
H	2.367183000	1.568488000	6.963939000
C	-0.089575000	4.656559000	3.133417000
H	0.476333000	4.143271000	2.363102000
H	-1.067851000	4.904266000	2.723624000
H	0.421949000	5.600218000	3.333679000

cis-5a

52
Fe2C23N2H22SeO2

C	0.579215000	-0.083015000	0.566692000
Fe	2.035672000	0.825858000	1.229062000
Fe	-0.363012000	1.276583000	1.693561000
O	0.197511000	-0.930711000	-0.125274000
C	1.175653000	1.689077000	2.629130000
N	1.549787000	2.240518000	3.738935000
C	2.947726000	2.295773000	4.181137000
H	3.474436000	1.407791000	3.849283000
H	2.973944000	2.333273000	5.267788000
H	3.442477000	3.184333000	3.790365000
H	4.448486000	-0.434818000	0.336611000
C	3.886688000	0.483418000	0.242252000
C	3.987152000	1.605942000	1.103639000
H	2.606571000	0.092640000	-1.529122000
H	4.678814000	1.710663000	1.925129000
C	3.063730000	2.580709000	0.648518000
H	2.898431000	3.555660000	1.085591000
C	2.384248000	2.051593000	-0.467691000
H	1.624430000	2.555164000	-1.046188000
C	2.904831000	0.750595000	-0.726274000
N	2.300314000	-0.668532000	2.399347000
C	2.418401000	-1.552964000	3.143736000
Se	2.599144000	-2.899396000	4.288240000
C	-0.752309000	-0.017864000	2.826075000
O	-1.099028000	-0.835064000	3.537724000
H	0.021395000	3.844040000	0.436762000
C	-0.722940000	3.107101000	0.699012000
C	-1.465328000	3.055948000	1.906729000
H	-0.779964000	1.805889000	-1.104984000
H	-1.394403000	3.739769000	2.736625000
C	-2.338210000	1.951118000	1.833542000
H	-3.041734000	1.651891000	2.597916000
C	-2.150545000	1.313822000	0.573956000
H	-2.682739000	0.447110000	0.210565000
C	-1.159480000	2.040040000	-0.120201000
C	0.607093000	2.834333000	4.649381000
C	-1.214150000	3.979475000	6.368668000
C	0.308334000	4.191216000	4.506211000
C	0.080076000	2.057692000	5.678890000
C	-0.855992000	2.651767000	6.524280000
C	-0.621419000	4.748066000	5.376705000
H	-1.294734000	2.062453000	7.320391000
H	-0.883604000	5.793924000	5.274305000
H	-1.945443000	4.423409000	7.031665000
C	0.977618000	5.035019000	3.461373000
H	2.031830000	5.192525000	3.698034000
H	0.507575000	6.014712000	3.397462000
H	0.941418000	4.579953000	2.472259000
C	0.541511000	0.656457000	5.956717000
H	1.039266000	0.186034000	5.112759000
H	-0.290270000	0.021384000	6.258305000
H	1.253869000	0.662125000	6.784560000

trans-6a

51
Fe2C23N2H22O2

C	0.606625000	-0.252985000	0.741580000
Fe	1.878747000	0.910026000	1.266102000
Fe	-0.470031000	0.808974000	2.182062000
O	0.250910000	-1.109042000	0.047938000
C	1.042725000	1.702499000	2.724236000
N	1.453612000	2.445913000	3.708256000
C	2.855418000	2.841220000	3.882951000
H	3.509603000	1.989276000	3.723333000
H	3.001983000	3.208439000	4.895152000
H	3.115174000	3.641971000	3.192518000
H	0.012869000	-0.319879000	4.805326000
C	-0.718147000	-0.229407000	4.017884000
C	-0.888799000	-1.139697000	2.957845000
H	-1.857429000	1.625338000	4.550041000
H	-0.286272000	-2.020231000	2.787836000
C	-1.970547000	-0.696644000	2.152792000
H	-2.359007000	-1.185377000	1.272125000
C	-2.476172000	0.486705000	2.742726000
H	-3.310884000	1.069001000	2.376575000
C	-1.698541000	0.792013000	3.882692000
H	4.319785000	-0.008858000	0.102124000
C	3.618347000	0.811398000	0.066954000
C	3.649809000	1.969426000	0.887405000
H	2.213056000	0.185274000	-1.531535000
H	4.408591000	2.199612000	1.618494000
C	2.548386000	2.784907000	0.526845000
H	2.298200000	3.747101000	0.951199000
C	1.830522000	2.127356000	-0.492898000
H	0.954645000	2.500713000	-1.001459000
C	2.502703000	0.906391000	-0.781397000
C	-1.017663000	2.061248000	1.073122000
O	-1.442029000	2.846075000	0.362656000
C	2.561864000	-0.357097000	2.496087000
N	2.982734000	-1.118737000	3.266099000
C	0.563209000	2.917350000	4.733278000
C	-1.144117000	3.773296000	6.726006000
C	-0.277490000	3.999010000	4.479666000
C	0.610195000	2.288925000	5.982558000
C	-0.268828000	2.725257000	6.967543000
C	-1.136708000	4.409638000	5.497356000
H	-0.261097000	2.238720000	7.935461000
H	-1.802637000	5.245983000	5.320242000
H	-1.822112000	4.102156000	7.503013000
C	1.597056000	1.201955000	6.300443000
H	2.517162000	1.627101000	6.708041000
H	1.881284000	0.607579000	5.432993000
H	1.194015000	0.527940000	7.055031000
C	-0.299497000	4.737367000	3.174494000
H	0.553265000	4.516096000	2.537000000
H	-1.203325000	4.504144000	2.611286000
H	-0.309131000	5.812630000	3.354058000

Se_8

8
Se8

Se	1.433625000	1.823713000	-1.312570000
Se	-0.471627000	2.646264000	-0.283578000
Se	0.724196000	-0.002694000	-2.551903000
Se	-0.466249000	-2.646721000	-0.280111000
Se	1.441666000	-1.821177000	-1.305501000
Se	-0.418386000	-1.849882000	1.896674000
Se	-0.422478000	1.852747000	1.894833000
Se	-1.820746000	-0.000303000	1.942154000
